# PIR-SBFR: A Calibration-Free Sensor-to-Inference Routing Interface for Aerial Optical Object Detection

**DOI:** 10.3390/s26185985

**Published:** 2026-09-21

**Authors:** Zixin Wang, Jingchao Liu, Zizheng Zhao, Xiaoyu Dong, Zhirui Xue, Junhao Hu, Chengxin Zhu

**Affiliations:** School of Computer Science, Xijing University, Xi’an 710123, China; a137762431@gmail.com (Z.W.); 2508540402019@stu.xijing.edu.cn (Z.Z.); 2508540402014@stu.xijing.edu.cn (X.D.); 2508540402010@stu.xijing.edu.cn (Z.X.); 2508540402015@stu.xijing.edu.cn (J.H.); 2508540402033@stu.xijing.edu.cn (C.Z.)

**Keywords:** optical image sensing, acquisition-aware perception, object detection, ground sampling distance, image-quality descriptors, controlled observation descriptors, multiscale feature routing, aerial optical imaging

## Abstract

**Highlights:**

**What are the main findings?**

- Nominal feature-map resolution can diverge from recoverable evidence as optical acquisition quality changes.
- PIR-SBFR provides a calibration-free sensor-to-inference routing interface that reconciles scene demand with an ordered reliability prior.

**What are the implications of the main findings?**

- Ten paired runs improve AP by 2.54 and 2.52 percentage points on DIOR and AI-TOD-v2, respectively.
- Matched tiny-object comparisons, three host detectors, controlled quality tests, and Jetson measurements demonstrate accuracy gains and embedded inference feasibility.

**Abstract:**

Aerial detection requires fine detail for small targets and broad context for large structures. Sampling loss, blur, and noise can weaken detail without changing feature-map resolution, creating a demand–reliability mismatch. We present Physical Imaging Reliability-Guided Scale-Biased Feature Reweighting (PIR-SBFR), a sensor-to-inference interface that separates scene-scale demand from observation reliability. A visual branch estimates demand, while a fixed analytic rule converts relative sampling, sharpness, noise, and field availability into an ordered multiscale prior. The routing rule requires no sensor-specific response fitting. On public DIOR images, ten paired runs increase average precision (AP) from 62.98% (SD 0.32) to 65.52% (SD 0.40). On the study-specific AI-TOD-v2 split, AP rises from 29.58% (SD 0.27) to 32.10% (SD 0.41). Matched local reproductions of three contemporary tiny-object detectors reach 30.42–31.59% AP under the same protocol. PIR-SBFR also improves three compatible host detectors by 2.09–2.54 percentage points. All 27 controlled quality conditions and nine held-out degradation types favor PIR-SBFR. On a Jetson Orin NX, its FP16 end-to-end median latency is 19.55 ms. These results demonstrate effective acquisition-conditioned routing under controlled observation changes on public aerial imagery.

## 1. Introduction

Intelligent optical sensing couples image formation, digital acquisition, and neural inference in one operational chain. The spatial information available for downstream recognition is constrained before the detector receives a tensor. Sampling footprint, optical transfer, noise, altitude, and viewing geometry alter how much spatial evidence survives. In aerial imagery, a vehicle may occupy only a few uncertain pixels, while a bridge or airport still requires broad context [1,2,3,4,5,6,7,8]. The sensing problem extends beyond object-scale variation to variation in the reliability of the evidence available at each scale.

Feature pyramids expose several tensor resolutions, but tensor resolution is not equivalent to recoverable image detail. P3 remains geometrically fine after coarse sampling or blur has removed the edges that would make it informative. In aerial imaging, acquisition geometry and sensor sampling determine ground sampling distance (GSD), which summarizes the projected ground footprint. A modulation transfer function/point-spread function (MTF/PSF) descriptor represents spatial-detail transfer. A signal-to-noise ratio (SNR) descriptor represents the stability of weak contrast. These acquisition variables constrain the observation upstream of the network.

This distinction creates a demand–reliability mismatch. Scene content determines the scale needed for recognition and localization. The sensing process determines whether evidence at that scale remains reliable. We use scene-scale demand for the first quantity and observation-dependent reliability for the second. The central hypothesis is that multiscale detection improves when content-driven routing is constrained by a monotonic acquisition-reliability signal.

Existing multiscale detectors primarily infer utility from visual activations. You Only Look Once (YOLO) variants preserve fine maps, add detection heads, or introduce attention [4,5,9]. Feature Pyramid Network (FPN), Bidirectional Feature Pyramid Network (BiFPN), Adaptively Spatial Feature Fusion (ASFF), and Asymptotic Feature Pyramid Network (AFPN) improve cross-level exchange through learned paths or weights [10,11,12,13]. Such methods can learn useful responses to degraded images. However, a weak P3 activation alone does not uniquely distinguish low fine-scale demand from lost fine-scale evidence. Acquisition descriptors supply an additional source of information about this ambiguity, rather than replacing visual inference.

Acquisition-aware detection provides a complementary direction. GSDDet uses spatial-resolution guidance, Meta-YOLO introduces platform telemetry, and feature-wise linear modulation (FiLM) supplies general conditioning [14,15,16]. Naturally occurring distribution shifts further expose the sensitivity of visual inference to observation changes [17]. Unrestricted conditioning, however, does not specify how sampling, blur, or noise should affect P3–P5. It may learn platform, location, or scene correlations unrelated to image reliability. Incomplete records introduce another ambiguity unless missing fields have an explicit neutral state.

Physical Imaging Reliability-Guided Scale-Biased Feature Reweighting (PIR-SBFR) addresses this gap as a calibration-free sensor-to-inference routing interface. It separates a scale-supervised estimate of scene demand from an ordered reliability prior based on GSD, MTF/PSF-related sharpness, SNR, and field availability. The visual branch determines which scales the scene requires; the analytic branch constrains their relative reliability under the specified observation conditions. Neither source is sufficient alone: acquisition quality does not reveal object composition, and scene content does not uniquely identify acquisition quality. Logit-space reconciliation combines both sources before fusion. For fixed visual demand, the analytic contribution prescribes how worsening acquisition conditions change finer-to-coarser allocation odds. Availability masking defines the neutral missing-field state, metadata dropout exposes it during training, and a P5 bypass retains coarse evidence outside normalized allocation.

Here, calibration-free routing means that the descriptor-to-reliability rule is fixed without fitting a response function to a particular camera or sensor. Descriptor acquisition may require upstream characterization, measurement, or a separately validated estimator. The public-benchmark experiments use descriptors assigned from controlled operators or nominal reference labels rather than native sensor telemetry. This setup evaluates the routing interface under specified observation changes.

PIR-SBFR is evaluated against a same-study visual-only control with an identical backbone, shared fusion path, and prediction head. The full comparison includes the routing module and its associated augmentation and training terms. Component controls examine subsets of these changes. Cross-host evaluation separates the routing gain from the contribution of the internal architecture.

Evaluation combines two public detection benchmarks with controlled observation-quality tests. Same-code controls and factorial ablation examine competing architectural explanations, while descriptor interventions, routing probes, and held-out degradations evaluate correspondence, internal behavior, and performance outside the development operators.

Across ten paired runs, PIR-SBFR improves average precision (AP) by 2.54 percentage points (pp) on the Dataset for Object Detection in Optical Remote Sensing Images (DIOR). AP improves by 2.52 pp on the Aerial Images Tiny Object Detection Dataset, version 2 (AI-TOD-v2), under the study-specific split. Matched local reproductions of three contemporary tiny-object detectors provide a common-protocol comparison on AI-TOD-v2. Three compatible host detectors test whether the interface depends entirely on the internal architecture. PIR-SBFR also outperforms the internal visual-only baseline in the 27 controlled quality conditions and nine held-out degradations. The latter yield a mean gain of 4.37 pp. Jetson measurements evaluate the inference path beyond desktop graphics processing unit (GPU) timing.

The contributions of this study are threefold:

(1) We formulate the demand–reliability mismatch in multiscale visual inference. The formulation distinguishes the scale required by scene content from the evidence supported by the acquired observation.
(2) We formulate PIR-SBFR as a calibration-free sensor-to-inference routing interface. The interface factorizes P3–P5 allocation into scale-supervised scene demand and an ordered acquisition-reliability prior. For fixed visual demand, each available degradation coordinate monotonically lowers finer-to-coarser allocation odds.
(3) We evaluate the proposed factorization through ten-run endpoint comparisons, matched tiny-object baselines, three compatible host detectors, descriptor interventions, routing probes, controlled observation-quality conditions, held-out degradations, and embedded inference. These tests examine accuracy, allocation behavior, descriptor correspondence, architectural dependence, and deployment cost.

**Figure 1:**
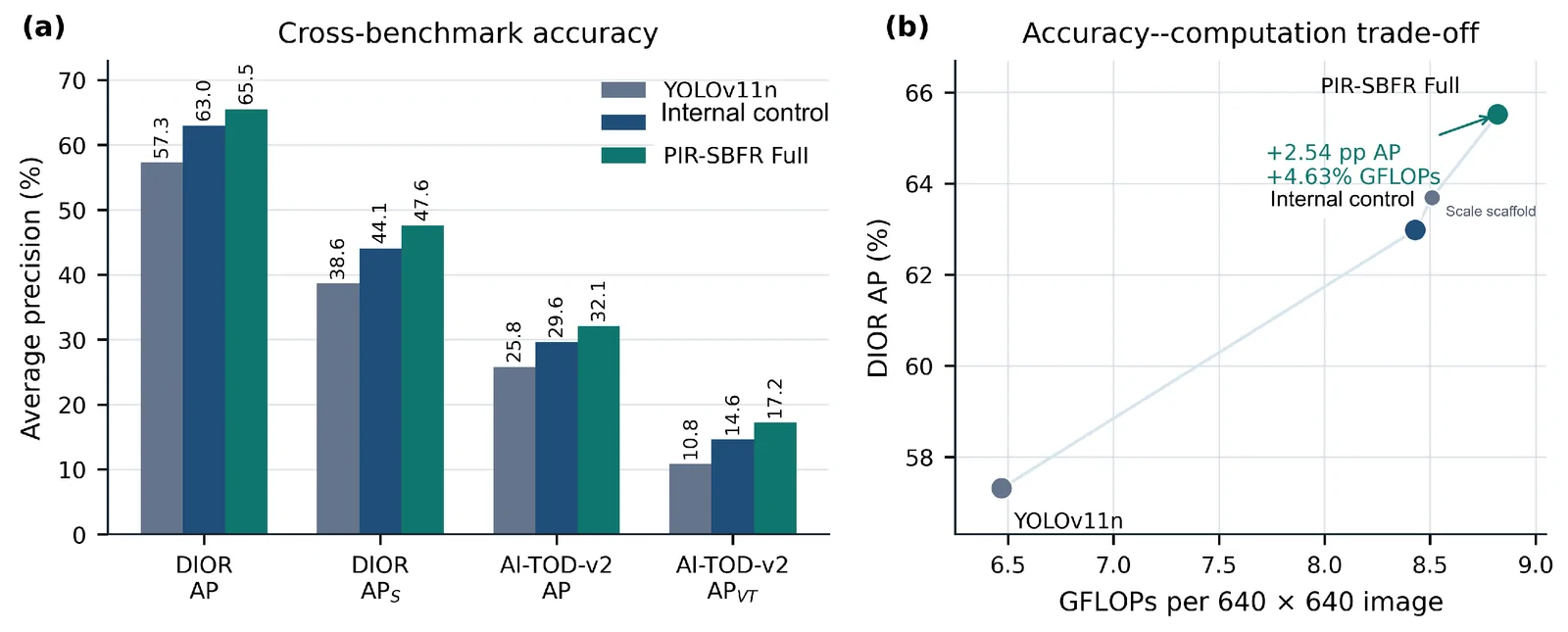
Overview of benchmark accuracy and computation. (**a**) AP and small/tiny-object AP on DIOR and the study-specific AI-TOD-v2 split. Internal-baseline and PIR-SBFR bars are ten-run means. The stock-YOLOv11n bars reproduce the retained three-seed architecture-reference summaries: 57.31/38.64% on DIOR and 25.82/10.84% on AI-TOD-v2. (**b**) DIOR AP versus GFLOPs for YOLOv11n, the internal baseline, the visual-demand/P5-preservation control (“Scale scaffold”), and PIR-SBFR Full. The scale-scaffold AP is its retained three-seed A2 mean; internal-baseline and PIR-SBFR AP are ten-run means. Resource values are single standardized profiles. Section 4.9 separately reports embedded end-to-end measurements.

Figure 1 previews the principal benchmark accuracy and computational-cost endpoints. It places the cross-dataset accuracy gains beside the measured computation increase. Section 3 defines the evaluation protocol, and Section 4 presents the mechanism-specific analyses.

## 2. Related Work

### 2.1. Image Formation and Sensor-Supported Evidence

An optical image is jointly determined by scene radiance, sampling geometry, spatial transfer, detector noise, and digitization. MTF or spatial-frequency response characterizes how an imaging system transfers contrast across spatial frequencies. SNR describes the stability of the measured signal relative to sensor noise [18,19,20]. In aerial imaging, GSD additionally summarizes the projected sampling footprint. These quantities do not determine recognition accuracy directly, but they do bound the evidence delivered to a perception model.

Image-quality degradation is consequential for neural inference. Blur, noise, pixelation, and compression reduce the accuracy of otherwise strong visual models [21]. Motion blur also increases localization error in online object detection [22]. Corruption-specific augmentation can improve robustness, but performance may remain sensitive to unseen distortions [23]. This evidence motivates a downstream model that distinguishes feature-map geometry from acquisition-supported information.

### 2.2. Multiscale Processing of Sensor-Captured Images

FPN combines semantic and spatial information through top-down and lateral paths [10]. BiFPN learns normalized weights over repeated bidirectional connections [11]. ASFF predicts spatially varying scale weights [12]. AFPN progressively merges adjacent levels to reduce semantic gaps [13]. Related aerial-image detectors preserve fine maps, enlarge contextual support, or add attention for small and elongated objects [4,5,6,7,8].

More recently, UAV-DETR combines spatial and frequency-domain fusion with detail-preserving downsampling and spatial alignment [24]. Its alignment offsets and fusion weights are learned from visual features. This approach addresses detail retention and cross-level misalignment, whereas PIR-SBFR additionally conditions scale allocation on supplied acquisition-quality descriptors.

These methods exchange visually derived representations without explicit acquisition-quality descriptors in their standard formulations. Resampling, blur, and noise can leave tensor dimensions unchanged while changing the information carried by those tensors. A weak fine-level response may indicate an empty region or an unresolved target. Detection loss alone does not uniquely identify the cause. Content-driven fusion can learn corruption robustness, but it does not explicitly distinguish scene demand from the reliability constraints represented by acquisition descriptors.

### 2.3. Contemporary Tiny-Object Detection

Tiny-object detectors increasingly adapt query allocation to crowded scenes. DQ-DETR uses dynamic queries to address object-count variation [25]. D3Q develops density-aware query generation and a two-stage training strategy [26]. Dome-DETR jointly manipulates features and queries using density information [27]. These methods provide task-specific context beyond comparisons with compact general-purpose YOLO models.

Density-guided query allocation and acquisition-reliability routing address related but different constraints. The former adapts detection capacity to the number and arrangement of tiny objects. PIR-SBFR introduces supplied observation descriptors to constrain the reliability of pyramid evidence. Section 4.1.2 compares local reproductions under a common split, input size, and evaluator.

### 2.4. Acquisition-Aware and Metadata-Conditioned Perception

Acquisition descriptors provide context that red–green–blue (RGB) activations may not identify uniquely. GSDDet guides aerial-image detection with ground sampling distance [14]. Meta-YOLO uses platform telemetry to construct spatial metadata maps that guide deformable-convolution sampling [15]. Its metadata-conditioned spatial sampling differs from PIR-SBFR’s ordered allocation across pyramid levels. FiLM supplies a general mechanism for feature-wise conditioning [16]. WILDS documents failures under naturally occurring distribution shifts, including changes in satellite imagery [17].

The 2025 condition-aware PCDF framework uses acquisition and environmental cues to guide RGB–infrared fusion [28]. It learns condition prompts and channel-level modality weights; its inference path does not require explicit condition annotations. This differs from supplying available quality descriptors to a fixed monotonic P3–P5 prior. PCDF addresses modality allocation, while PIR-SBFR separates scene-scale demand from acquisition reliability within an RGB feature pyramid. Both use observation context, but their input assumptions and allocation targets differ.

Freely learned conditioning does not guarantee an acquisition-consistent response. An unrestricted embedding may encode location, platform, or scene associations unrelated to observation quality. It also does not specify how reduced sampling, optical transfer, or SNR should change P3–P5 allocation. Missing fields require a state distinct from a valid zero. These issues motivate a constrained interface between acquisition descriptors and feature routing.

### 2.5. Domain Generalization and Acquisition-Conditioned Inference

Domain-generalized object detection aims to retain detection performance on unseen target domains without access to target-domain data during training. Single-source approaches can diversify source images and align predictions across augmented views, as illustrated by Danish et al. [29]. Such training reduces dependence on source appearance without requiring acquisition descriptors for each test image.

PIR-SBFR addresses a different input setting: it uses available observation descriptors during inference and falls back to visual routing when they are missing. Its main intervention is a condition-dependent allocation rule, not the learning of domain-invariant features. Paired augmentation and prediction consistency overlap with robustness-oriented training, but the analytic descriptor path is an additional assumption and information source.

### 2.6. Spatial Context and Inter-Channel Relationships

Feature reliability also depends on relationships within a representation, not only on its spatial scale. In hyperspectral anomaly detection, the sliding dual-window-inspired reconstruction network of Wang et al. uses surrounding context to reconstruct a masked center region [30]. Excluding the central signal encourages reconstruction of background context instead of reproduction of anomalous spectra. SPG-OD uses spectral priors and grouped local spectral enhancement to improve object–background discrimination in hyperspectral imagery [31].

These studies motivate attention to spatial context and spectral dependencies, but their observation model differs from the RGB setting evaluated here. Hyperspectral bands represent wavelength-indexed measurements; learned feature channels need not have that interpretation. PIR-SBFR allocates evidence across pyramid levels and retains a shared content-based channel mapping downstream.

### 2.7. Separating Scene Demand from Acquisition Reliability

Prior work addresses three related questions: how to preserve spatial detail, how to fuse multiscale features, and how to condition perception on acquisition context. These questions are commonly handled in separate formulations. Content-driven fusion estimates feature utility from visual tensors, whereas generic metadata conditioning permits external variables to modify a network without prescribing a reliability direction. The cited formulations do not explicitly separate scene-required scale from acquisition-supported evidence.

The specific gap is a routing formulation that distinguishes an object’s scale requirements from the observation conditions supporting those requirements. FPN and BiFPN specify feature-exchange paths and fusion weights, while ASFF learns spatial allocation from visual features. GSD guidance and generic metadata conditioning introduce acquisition context, but the cited formulations do not jointly provide scale-targeted visual demand and the ordered three-descriptor reliability rule considered here.

PIR-SBFR combines these two quantities explicitly. The visual source is supervised by scene-scale counts; the analytic source maps available GSD, sharpness, and SNR descriptors to an ordered P3–P5 prior. Their logit-space reconciliation gives a prescribed derivative sign for pairwise allocation odds when visual demand is fixed. Availability masking supplies a neutral state, and the P5 bypass preserves an unweighted coarse route. Together, these properties define the demand–reliability decomposition.

This factorization produces testable mechanism expectations. Reduced observation quality should move allocation away from P3 in the direction specified by the ordered prior. Breaking image–descriptor correspondence should reduce the benefit on otherwise unchanged images, while masking every descriptor should return the analytic source to a neutral state without disabling the detector. Table 1 summarizes the corresponding distinctions, and Section 3 describes the mechanism-centered evaluation.

## 3. Materials and Methods

### 3.1. Problem Formulation: Feature Resolution and Sensor-Supported Evidence

Let *I* denote an image delivered by an optical imaging system, m a low-dimensional descriptor of its observation conditions, and a the corresponding field-availability mask. Let $\hat{Y}$ collect the predicted classes, confidence scores, and bounding boxes. The descriptors provide acquisition context; they are not an independent sensing modality. The shared feature extractor produces $P_{3}$, $P_{4}$, and $P_{5}$ at strides 8, 16, and 32. P3–P5 denote pyramid levels, whereas $P_{3}$–$P_{5}$ denote their feature tensors. These tensors form the visual side of the sensor-to-inference interface: network geometry fixes their dimensions, whereas the acquired observation partly determines their evidential value.

The PIR-SBFR routing ablations share the same feature extractor, Feature Pyramid Network–Path Aggregation Network (FPN–PAN) fusion path, and prediction mapping. The visual-only control routes $P_{3}$–$P_{5}$ from image content alone, whereas PIR-SBFR adds acquisition descriptors and availability masks at the routing interface. The Dilated Receptive Field Block (DRFB) and Feature-Aware Coupled Head (FACH) remain fixed within these internal routing comparisons and are not treated as sensing contributions. Stock YOLOv11n and alternative fusion architectures are separate reference configurations. The forward mapping of the full configuration is

(1) $$\left\{P_{3},P_{4},P_{5}\right\}=B_{\mathrm{DRFB}}\left(I\right),\;\left\{F_{3},F_{4},F_{5}\right\}=N_{\mathrm{PIR}}\left(\left\{P_{i}\right\},m,a\right),$$

(2) $$\hat{Y}=H_{\mathrm{FACH}}\left(F_{3},F_{4},F_{5}\right).$$

Here, $B_{\mathrm{DRFB}}$ and $H_{\mathrm{FACH}}$ are shared upstream and downstream mappings. The only condition-dependent interface is $N_{\mathrm{PIR}}$, which combines visual scene demand with descriptor-derived reliability. Their reconciliation produces one normalized allocation over $P_{3}$–$P_{5}$. The defining property is the constrained route by which acquisition descriptors enter inference.

**Figure 2:**
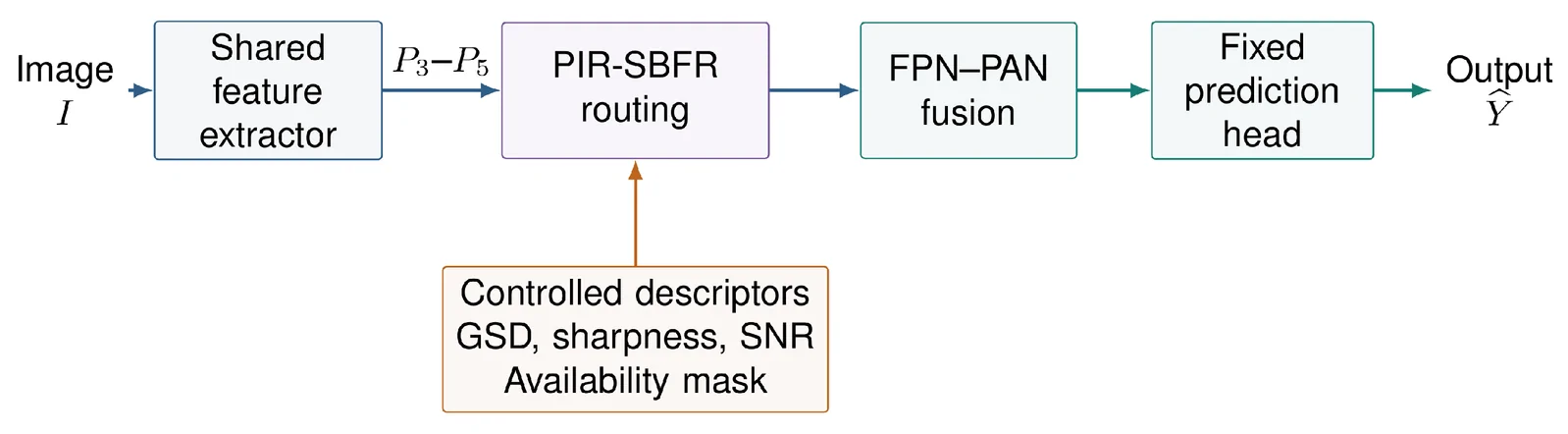
Location of PIR-SBFR within the shared detection path. Blue arrows carry image features; the orange arrow supplies controlled observation descriptors and their availability mask. The feature extractor and prediction head remain fixed across controlled configurations. Section 3.3 expands the two-source routing calculation and the P5 bypass, which is omitted from this overview. GSD: ground sampling distance; SNR: signal-to-noise ratio.

Figure 2 locates this interface within the shared inference path. An unweighted projection preserves access to P5 outside the normalized allocation, reducing the risk that strong fine-scale demand suppresses broad structural evidence.

### 3.2. Shared Feature-Extraction Path

DRFB provides the shared feature representation for the visual-only control and PIR-SBFR. It receives image features and remains fixed across routing configurations. For an input tensor $X^{\left(l-1\right)}\in R^{H\times W\times C}$, a 1×1 convolution followed by batch normalization (BN) first reduces the channel dimension:

(3) $$X_{\mathrm{red}}^{\left(l\right)}=\sigma \;\left(\mathrm{BN}\;\left({\mathrm{Conv}}_{1\times 1}^{C\to C/2}\left(X^{\left(l-1\right)}\right)\right)\right),$$

where σ is the activation function. Two 3×3 convolutions with dilation rates 2 and 3 process the reduced tensor without further downsampling:

(4) $$D_{r}^{\left(l\right)}=\sigma \;\left(\mathrm{BN}\;\left({\mathrm{DConv}}_{3\times 3}^{r}\left(X_{\mathrm{red}}^{\left(l\right)}\right)\right)\right),\;r\in \left\{2,3\right\}.$$

The two responses are concatenated and compressed to form $X_{\mathrm{ctx}}^{\left(l\right)}$. A final 1×1 projection restores *C* channels before the residual addition:

(5) $$X_{\mathrm{ctx}}^{\left(l\right)}={\mathrm{Conv}}_{\mathrm{cat}}\;\left([D_{2}^{\left(l\right)},D_{3}^{\left(l\right)}]\right),$$

(6) $$X^{\left(l\right)}=X^{\left(l-1\right)}+\phi_{1\times 1}^{C/2\to C}\;\left(X_{\mathrm{ctx}}^{\left(l\right)}\right).$$

**Figure 3:**
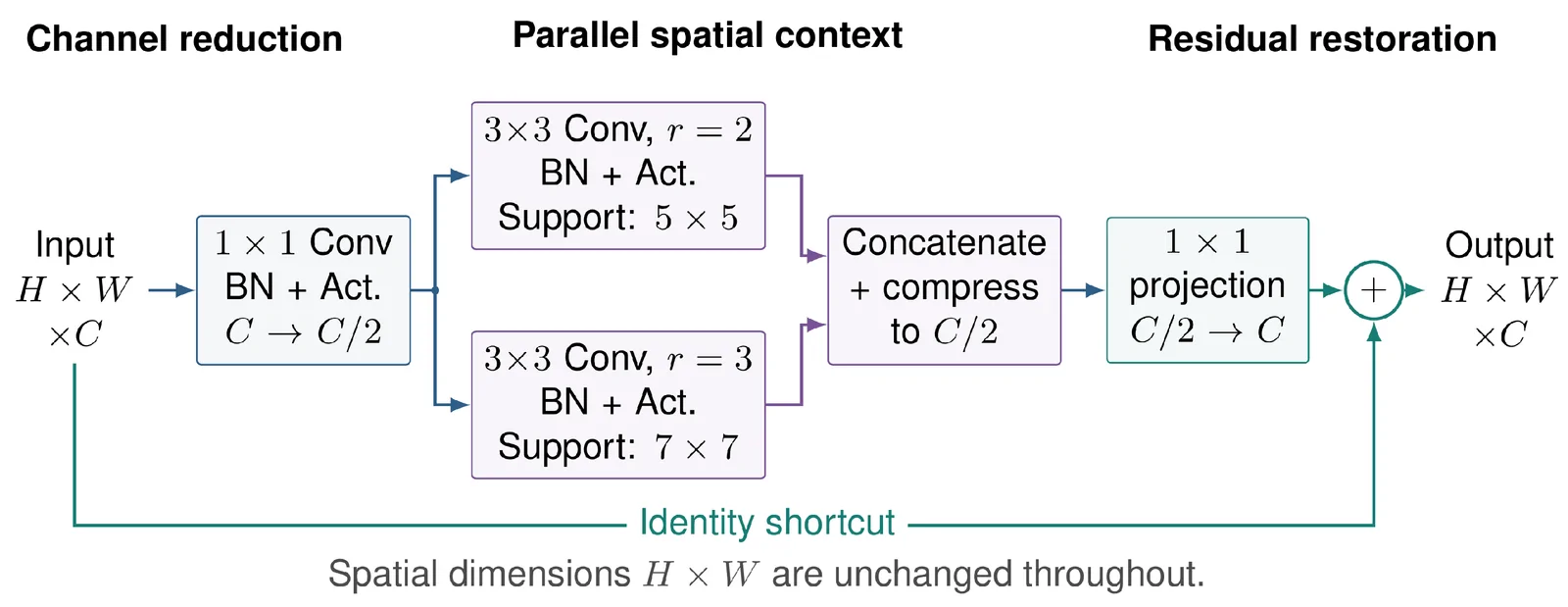
Shared Dilated Receptive Field Block (DRFB), held fixed across routing configurations. Dilation rates r=2 and r=3 give effective kernel supports of 5×5 and 7×7 on the input feature map. Concatenation, channel compression, and projection precede identity addition. Spatial dimensions remain unchanged, and no acquisition descriptor enters this block. BN: batch normalization; Act.: activation.

The identity and dilated paths provide a common context-preserving representation for every routing configuration. Dilated convolution expands the receptive field without reducing map size, and residual addition stabilizes information transfer [32,33]. Figure 3 documents this fixed extraction path.

### 3.3. PIR-SBFR as a Sensor-to-Inference Routing Interface

**Figure 4:**
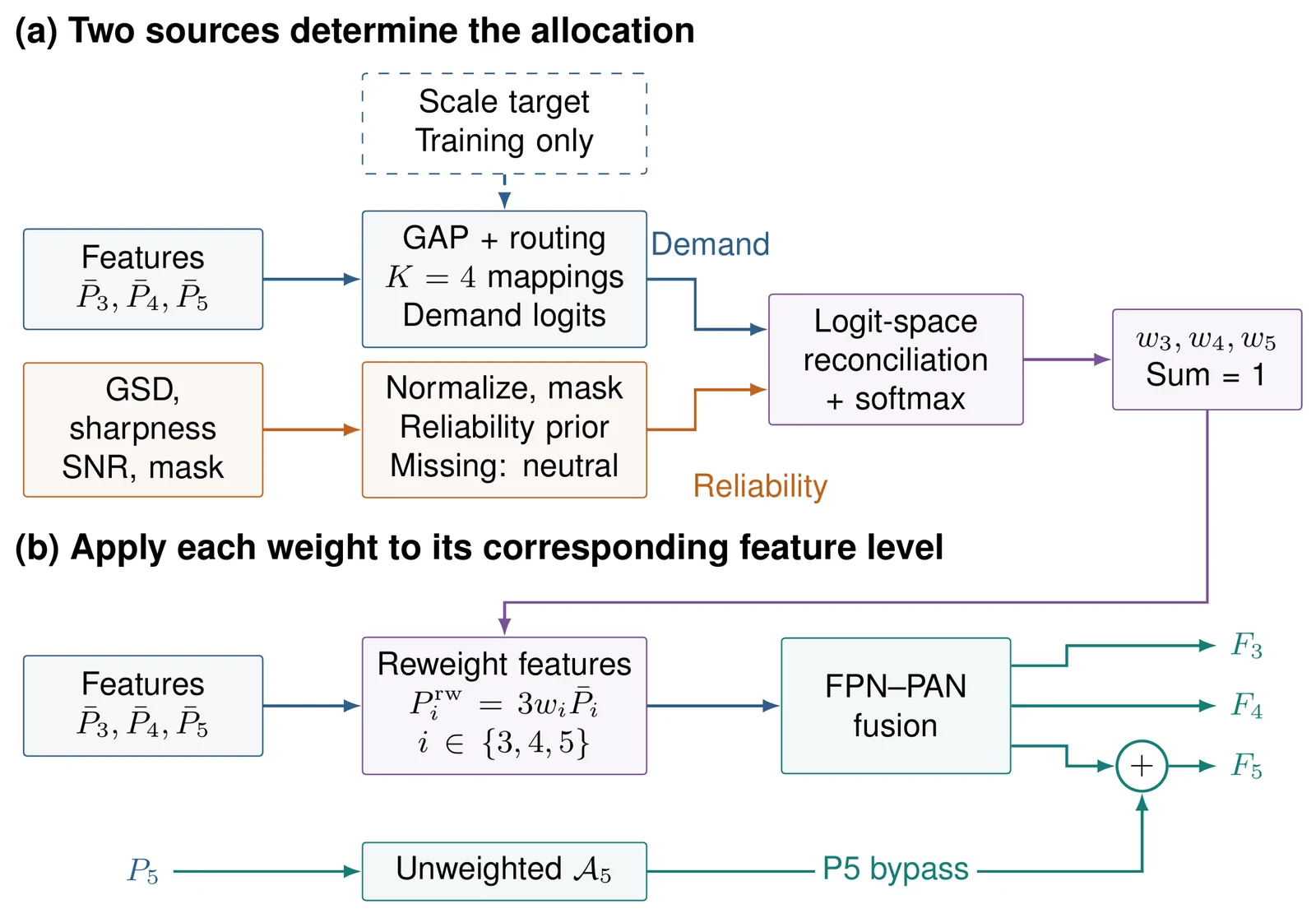
PIR-SBFR routing and feature paths. Aligned pyramid features provide visual demand and remain the tensors being reweighted. Controlled descriptors supply a separate analytic reliability prior. Reconciliation produces three scalar weights, each applied only to its corresponding level before FPN–PAN fusion. The unweighted P5 projection is added only to the coarse output. Solid arrows denote inference computations; the dashed arrow denotes training-only scale supervision. GAP: global average pooling.

PIR-SBFR connects two quantities that arise at different stages of the sensing chain. Visual features estimate the scale demanded by the scene, while acquisition descriptors define an ordered constraint on the evidence supported at each pyramid level. Logit-space reconciliation converts these signals into $P_{3}$–$P_{5}$ allocation weights before the shared downstream fusion path. Figure 4 summarizes this interface and the direct P5 preservation path.

#### 3.3.1. Acquisition Descriptors and Observation-Reliability Prior

For image *I*, the acquisition descriptor and its availability mask are

(7) $$m={\left[g,q,s\right]}^{T},\;a={\left[a_{g},a_{q},a_{s}\right]}^{T}\in {\left\{0,1\right\}}^{3}.$$

Let *G* denote a physical ground sampling distance and $G_{0}$ a compatible reference footprint. The dimensionless descriptor is $g=G/G_{0}$, with normalized reference $g_{\mathrm{ref}}=1$. In the controlled experiments, g=2 or g=3 denotes a twofold or threefold increase in effective sampling pitch relative to the resized input.

For the isotropic Gaussian operator, the scalar sharpness descriptor is the nominal normalized MTF at Nyquist, $q=\mathrm{MTF}(f_{N})$, where $f_{N}=0.5$ cycles per pixel. With Gaussian standard deviation σ in pixels, the ideal continuous model gives

(8) $$q=\exp\left(-2\pi^{2}\sigma^{2}f_{N}^{2}\right)=\exp\left(-\pi^{2}\sigma^{2}/2\right).$$

The descriptor *q* follows this ideal continuous model; sampling and kernel truncation can alter the realized MTF. Unmodified images receive the nominal reference label $q_{\mathrm{ref}}=0.50$.

The noise descriptor *s* is the nominal SNR target in decibels for the controlled noise operator. The binary variable $a_{j}$ equals one when field *j* is available and compatible with the stated convention. Missing fields are not inferred from class or bounding-box annotations. An arbitrary sharpness score or camera gain cannot be substituted for *q* or *s* without a validated conversion.

Descriptors encode the controlled intervention, with nominal reference labels for unmodified views. No image-based descriptor estimator is used. The native optical transfer and noise of the source images are unknown. Table 2 summarizes the descriptor definitions and handling.

The three descriptors represent different parts of image formation and use different units. We map them to non-negative degradation coordinates relative to $g_{\mathrm{ref}}=1$, $q_{\mathrm{ref}}=0.50$, and $s_{\mathrm{ref}}=30$ dB. These values define the controlled reference state, at which each degradation coordinate is zero:

(9) $$d_{g}=\max\;\left(0,\log\frac{g}{g_{\mathrm{ref}}}\right),\;d_{q}=\max\;\left(0,1-\frac{q}{q_{\mathrm{ref}}}\right),\;d_{s}=\max\;\left(0,\frac{s_{\mathrm{ref}}-s}{s_{\mathrm{ref}}}\right).$$

Each coordinate is zero at or above its reference quality and increases with the corresponding degradation. Let $d={\left[d_{g},d_{q},d_{s}\right]}^{T}$. The reliability prior for pyramid level i∈{3,4,5} is

(10) $$\rho_{i}^{\mathrm{phy}}=\exp\;\left[-\kappa_{i}^{T}\left(a⊙d\right)\right].$$

The non-negative decay vector $\kappa_{i}$ is fixed by pyramid stride $r_{i}\in \left\{8,16,32\right\}$:

(11) $$\kappa_{i}=\frac{8}{r_{i}}{\left[1,1,1\right]}^{T},\;\left[\begin{array}{c} \kappa_{3}^{T}\\ \kappa_{4}^{T}\\ \kappa_{5}^{T} \end{array}\right]=\left[\begin{array}{ccc} 1 & 1 & 1\\ 0.5 & 0.5 & 0.5\\ 0.25 & 0.25 & 0.25 \end{array}\right].$$

Decay is strongest at P3 and weakest at P5. This ordering is a monotonic observation-quality assumption used by the router. It represents the greater vulnerability of fine spatial evidence to sampling loss, blur, and noise. The inverse-stride rule sets the P3 coefficient to one and halves it at each coarser level. Equal coefficients across the normalized coordinates simplify the fixed analytic mapping.

The availability mask can disable each term independently. The reported clean-benchmark ablation isolates MTF/PSF and SNR because GSD remains at its reference. If every field is missing, a=0 and $\rho^{\mathrm{phy}}=1$. The analytic branch then contributes a neutral log prior.

#### 3.3.2. Scale-Ordering Assumption

The prior ranks relative feature reliability under the component-wise ordering $\kappa_{3j}>\kappa_{4j}>\kappa_{5j}$, for j∈{g,q,s}. This assumption represents the greater vulnerability of fine-scale evidence to the modeled degradations. The fixed inverse-stride coefficients implement this ordering, while availability masks remove unsupported fields. The controlled grid and routing probes test the resulting allocation direction, and descriptor perturbation and shuffling test image–descriptor correspondence.

#### 3.3.3. Visual Estimation of Scene-Scale Demand

The analytic prior limits trust but cannot determine what the scene requires. A separate visual path estimates demand from the feature pyramid. Each level is aligned to a common channel dimension and processed by global average pooling (GAP):

(12) $${\bar{P}}_{i}=A_{i}\left(P_{i}\right),\;z=\left[\mathrm{GAP}\left({\bar{P}}_{3}\right);\mathrm{GAP}\left({\bar{P}}_{4}\right);\mathrm{GAP}\left({\bar{P}}_{5}\right)\right].$$

A lightweight content-conditioned mapping with *K* transformations produces the residual routing logits:

(13) $$\pi =\mathrm{softmax}\left(W_{\pi }z\right),\;\delta^{\mathrm{vis}}=\sum_{k=1}^{K}\pi_{k}T_{k}\left(z\right),$$

where $T_{k}$ maps the pooled descriptor to three logits, and $\pi_{k}$ is its input-dependent mixture coefficient. The reported setting K=4 supplies four alternative learned mappings, each contributing to all three pyramid levels. The analytic reliability branch has no transformation bank. The visual branch estimates scene demand from image features. The visual estimate of the scene-scale distribution is

(14) $$\hat{q}=\mathrm{softmax}\left(\delta^{\mathrm{vis}}\right).$$

For an image containing $n_{S}$, $n_{M}$, and $n_{L}$ annotated instances, the target scale distribution is

(15) $$q=\frac{{\left[n_{S},n_{M},n_{L}\right]}^{T}+\epsilon }{n_{S}+n_{M}+n_{L}+3\epsilon }.$$

The small-, medium-, and large-object definitions follow Section 3.5.1. Their normalized counts supervise P3, P4, and P5, respectively. This scene-level target does not assign individual objects to specific detection heads. All three pyramid levels remain active in every forward pass. The auxiliary loss is the Kullback–Leibler (KL) divergence

(16) $$L_{\mathrm{scale}}=D_{\mathrm{KL}}\left(q\;∥\;\hat{q}\right).$$

Detection loss does not identify a unique internal allocation because different P3–P5 mixtures can produce similar endpoint losses. The auxiliary target anchors the visual logits to an observable property of each training image. It is intended to discourage a fixed dataset-level preference from masquerading as scene adaptation.

The target q is computed from annotations only during training. At inference, both $\hat{q}$ and $\delta^{\mathrm{vis}}$ are obtained from pyramid features.

With K=1, the visual mixture reduces to one learned mapping and its softmax coefficient is one. Increasing *K* adds candidate mappings but leaves the three-dimensional routing output unchanged. The branch separation and the directional property below do not depend on choosing four transformations. Section 3.5.5 reports the validation-only capacity sweep.

#### 3.3.4. Reliability-Aware Reconciliation in Logit Space

The final router treats demand and reliability as complementary evidence in logit space. For level *i*, the normalized allocation is

(17) $$w_{i}=\frac{\exp\;\left[(\delta_{i}^{\mathrm{vis}}+\eta \log\rho_{i}^{\mathrm{phy}})/\tau \right]}{\sum_{j=3}^{5}\exp\;\left[(\delta_{j}^{\mathrm{vis}}+\eta \log\rho_{j}^{\mathrm{phy}})/\tau \right]},\;\sum_{i=3}^{5}w_{i}=1.$$

The non-negative scalar η controls the ordered prior, and τ>0 sets routing temperature. Because the exponential prior is positive, its logarithm is well defined. The implementation evaluates $\log(\max\left(\rho_{i}^{\mathrm{phy}},\epsilon \right))$ to prevent numerical underflow. This clamp is inactive over the reported descriptor ranges.

For two distinct pyramid levels, define the pairwise allocation odds as

(18) $$O_{i\ell }=\frac{w_{i}}{w_{\ell }},\;i,\ell \in \left\{3,4,5\right\},\;i\neq \ell .$$

Values above one assign more normalized weight to level *i* than to level *ℓ*. These odds compare routing weights; they are not object-existence odds, class probabilities, or the one-versus-rest ratio $w_{i}/\left(1-w_{i}\right)$.

**Proposition** **1** (Directional monotonicity of the analytic routing contribution)**.** *For fixed visual logits and any levels i,ℓ∈{3,4,5}, Equation* (17) *gives*

$$\log\frac{w_{i}}{w_{\ell }}=\frac{\delta_{i}^{\mathrm{vis}}-\delta_{\ell }^{\mathrm{vis}}-\eta {\left(\kappa_{i}-\kappa_{\ell }\right)}^{T}\left(a⊙d\right)}{\tau }.$$

*For descriptor j∈{g,q,s}, its directional effect is*

$$\frac{\partial }{\partial d_{j}}\log\frac{w_{i}}{w_{\ell }}=-\frac{\eta a_{j}}{\tau }\left(\kappa_{ij}-\kappa_{\ell j}\right).$$

*If i is finer than ℓ, then $\kappa_{ij}>\kappa_{\ell j}$. Consequently, increasing an available degradation coordinate strictly reduces the finer-to-coarser allocation odds when η>0. A missing coordinate has $a_{j}=0$ and therefore has no analytic effect.*

**Proof.**  Taking pairwise log odds cancels the common softmax normalizer. Substitution of $\log\rho_{i}^{\mathrm{phy}}=-\kappa_{i}^{T}\left(a⊙d\right)$ gives the first expression. Differentiation with respect to $d_{j}$ gives the second. □

The guarantee isolates the analytic contribution at fixed visual logits. It does not require the end-to-end detector to remain monotonic when image degradation also changes visual features. The routing probes and controlled grid test whether the combined system follows the expected direction in the evaluated regimes.

Metadata influence remains auditable through $\rho_{i}^{\mathrm{phy}}$. Visual demand can counter a reliability prior that is too coarse for the scene. No unrestricted metadata embedding enters the feature tensor.

Consider two scenes with comparable small-object content. Fine sampling and good optical quality keep the P3 prior near one, so visual demand can favor P3. Coarser sampling or stronger blur reduces $\rho_{3}^{\mathrm{phy}}$ faster than $\rho_{4}^{\mathrm{phy}}$ and $\rho_{5}^{\mathrm{phy}}$. Allocation then moves toward contextual evidence. Under high image quality, broad structures can still favor P5 through visual demand. Soft normalization keeps all levels active.

The factor of three preserves the average feature scale when $w_{3}=w_{4}=w_{5}=1/3$:

(19) $$P_{i}^{\mathrm{rw}}=3w_{i}{\bar{P}}_{i},\;i\in \left\{3,4,5\right\}.$$

The standard FPN–PAN operations then fuse the reweighted levels:

(20) $$\left\{F_{3},F_{4},F_{5}\right\}=N_{\mathrm{FPN}-\mathrm{PAN}}\left(P_{3}^{\mathrm{rw}},P_{4}^{\mathrm{rw}},P_{5}^{\mathrm{rw}}\right).$$

Normalized allocation can reduce the contribution of P5 when fine-scale demand is high. We add an unweighted projection to the coarse output of the shared fusion stage:

(21) $$F_{5}\leftarrow F_{5}+A_{5}\left(P_{5}\right).$$

The bypass is intended to reduce the risk that fine-scale demand becomes a bottleneck for broad structural evidence. Its association with AP, ${\mathrm{AP}}_{S}$, and ${\mathrm{AP}}_{L}$ is examined through the paired 2×2 experiment.

#### 3.3.5. Inference with Incomplete Acquisition Records

Operational imaging systems and archived datasets do not always provide complete or synchronized acquisition-quality records. During training, PIR-SBFR masks each field independently with probability $p_{\mathrm{drop}}$. The analytic branch receives only a⊙d. Missingness contributes no degradation term and remains distinct from an observed numerical value.

If all fields are missing, $\rho^{\mathrm{phy}}=1$. The analytic log prior becomes constant across levels and cancels within the softmax. The detector then falls back to the scale-supervised visual route without retraining.

Training also pairs a clean view with a degraded view of the same source image. Section 3.5.3 specifies the training operators. Let $\left(p_{r},b_{r}\right)$ and $\left(p_{r}^{\prime },b_{r}^{\prime }\right)$ denote the class-probability vector and normalized box, respectively, for matched positive assignment *r*. The consistency term is

(22) $$L_{\mathrm{cons}}=\frac{1}{\left|R\right|}\sum_{r\in R}\left[D_{\mathrm{SKL}}\left(p_{r},p_{r}^{\prime }\right)+{∥b_{r}-b_{r}^{\prime }∥}_{1}\right],$$

where $D_{\mathrm{SKL}}$ denotes symmetric Kullback–Leibler divergence and R contains the matched positive assignments. The loss constrains predictions, while allowing routing weights to change because the two views have different feature reliability. The complete training objective is

(23) $$L=\frac{1}{2}\left(L_{\det}\left(I\right)+L_{\det}\left(I^{\prime }\right)\right)+\lambda_{\mathrm{scale}}L_{\mathrm{scale}}+\lambda_{\mathrm{cons}}L_{\mathrm{cons}}.$$

The first term averages detection loss over the paired views. The remaining terms constrain scene-scale demand and cross-view prediction stability. This objective permits routing to change with evidence quality while discouraging unstable endpoint predictions. Section 3.5.5 reports the fixed coefficients and training schedule.

### 3.4. Fixed Downstream Prediction Mapping

After observation-constrained allocation and shared fusion, a fixed downstream mapping produces classification and localization outputs. This mapping is identical across all controlled configurations and receives no acquisition descriptor. For fused output $F_{i}\in R^{H_{i}\times W_{i}\times C}$, global average pooling gives $z_{i}=\mathrm{GAP}\left(F_{i}\right)$. Channel reweighting is

(24) $$F_{i}^{c}=F_{i}⊙\sigma \left(W_{c}z_{i}+b_{c}\right).$$

Input-dependent coefficients $\phi_{i}=\mathrm{softmax}\left(W_{r}z_{i}+b_{r}\right)$ mix $K_{h}$ transformations. The head-bank size $K_{h}$ is distinct from the visual-router size *K*; the head is held fixed in the routing comparisons. An identity path remains outside the mixture:

(25) $$F_{i}^{\mathrm{out}}=F_{i}+\sum_{j=1}^{K_{h}}\phi_{ij}T_{j}\left(F_{i}^{c}\right).$$

A coupled convolution produces a shared representation before the two prediction branches separate:

(26) $$G_{i}=C_{\mathrm{coupled}}\left(F_{i}^{\mathrm{out}}\right),\;y_{i}^{\mathrm{cls}}=H_{\mathrm{cls}}\left(G_{i}\right),\;y_{i}^{\mathrm{reg}}=H_{\mathrm{reg}}\left(G_{i}\right).$$

**Figure 5:**
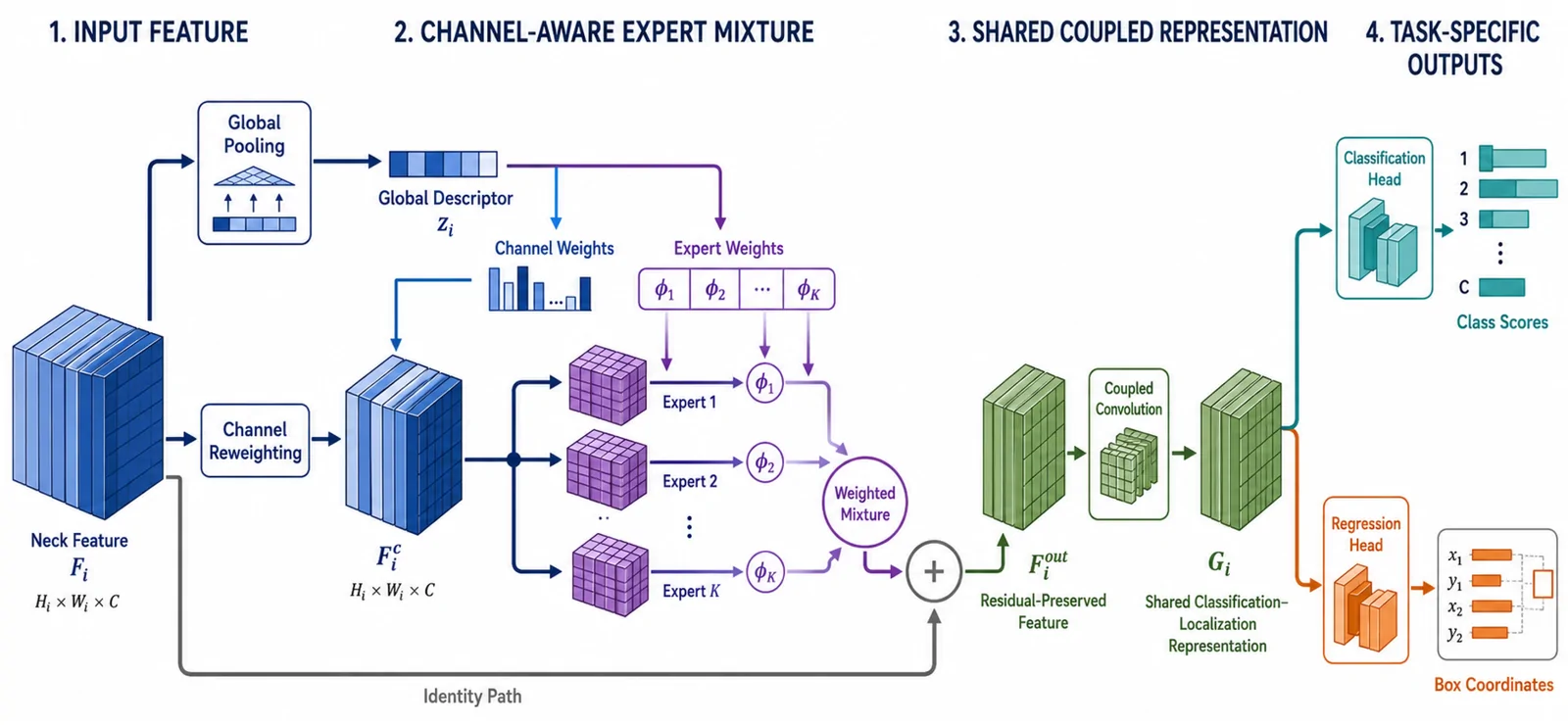
Fixed downstream prediction mapping used by the internal routing configurations. The Feature-Aware Coupled Head (FACH) receives fused features but no acquisition descriptors and separates into classification and regression outputs only after the shared representation. The schematic’s expert index *K* denotes the head-bank size $K_{h}$, not the visual-router-bank size.

The identity path retains the original response, and the shared tensor $G_{i}$ permits interaction between classification and localization before their final projections. Related input-dependent prediction mappings are described in [34,35,36]. Because FACH is fixed and metadata-free, the descriptor interventions evaluated later occur entirely upstream of this prediction mapping. Figure 5 documents the shared downstream computation.

### 3.5. Evaluation Protocol for Reliability-Aware Visual Sensing

#### 3.5.1. Datasets and Scale Definitions

The evaluation begins with two complementary scale regimes. DIOR tests whether the proposed allocation remains useful across broad scene and object scales. AI-TOD-v2 places the same mechanism near the tiny-object limit. DIOR contains 23,463 optical remote sensing images, 192,472 annotated instances, and 20 categories [2]. We use the official partitions without modification. The training, validation, and test partitions contain 5862, 5863, and 11,738 unique image identifiers. Every compared method uses these partitions.

Images are resized to 640×640 before bounding-box area is measured. Objects smaller than $32^{2}$ pixels are defined as small. Those from $32^{2}$ to less than $96^{2}$ pixels are medium, and those of at least $96^{2}$ pixels are large. These intervals define $n_{S},n_{M},n_{L}$, the scale-specific AP metrics, and the DIOR supervision target in Equation (15).

AI-TOD-v2 provides a second benchmark with substantially smaller objects. It contains 28,036 images, 752,754 instances, and eight categories, with a reported mean object size of 12.7 pixels [37]. The fixed study partitions contain 11,214 training, 5607 validation, and 11,215 test images. All methods run in this study use the same release and image identifiers.

AI-TOD-v2 defines very-tiny (2–8 pixels), tiny (8–16 pixels), small (16–32 pixels), and medium (32–64 pixels) intervals. For routing supervision, the very-tiny and tiny counts are combined for P3, while the small and medium counts supervise P4 and P5. Thus, $q_{\mathrm{AITOD}}=\mathrm{Normalize}\left(\left[n_{VT}+n_{T},n_{S},n_{M}\right]\right)$. Evaluation retains all four original intervals. Table 3 summarizes the partitions used throughout the study.

#### 3.5.2. Matched AI-TOD-v2, Cross-Host, and Channel-Gating Protocols

The matched AI-TOD-v2 comparison uses the same 11,214/5607/11,215 image identifiers, 640×640 inputs, 200 epochs, and ten seeds for every method. No method uses slicing, test-time augmentation, multiscale testing, ensembles, or extra training images. Each source image supplies the same clean and degraded views, and every detector averages its detection losses across both views. Only PIR-SBFR receives descriptors, scale supervision, and the consistency term.

Evaluation uses one COCO implementation, 1000 maximum detections per image, and the same very-tiny, tiny, small, and medium intervals. All methods are trained from scratch, and checkpoints are selected only by validation AP. The internal baseline and PIR-SBFR use stochastic gradient descent. The three transformer comparators retain AdamW and their architecture-specific learning-rate groups. Each method has a validation-search budget of at most four candidates. Comparator values are obtained from these local reproductions.

The backbone/remaining-parameter learning rates are $10^{-5}/\left(2\times 10^{-4}\right)$ for DQ-DETR, $10^{-5}/10^{-4}$ for DINO-D3Q, and $\left(2\times 10^{-5}\right)/\left(2\times 10^{-4}\right)$ for Dome-DETR-M. All three use weight decay $10^{-4}$ and batches of eight source images with two-step gradient accumulation, matching the effective batch of 16 used by the YOLO-derived configurations.

Cross-host evaluation adds the same PIR-SBFR interface to YOLOv11n, YOLOv11s, and the internal visual architecture. Projection widths follow the host channels, while *K*, η, τ, auxiliary-loss weights, and descriptor conventions remain fixed. Each baseline and PIR-SBFR pair uses the same ten seeds.

An exploratory channel-gating control uses the same DIOR validation protocol as the *K* sweep and five independent trainings per configuration. A content-only gate predicts channel weights from global pooled visual features. A descriptor-conditioned gate additionally receives g,q,s and the availability mask. Both variants retain the original P3–P5 router and differ only in the added gate. The analysis reports descriptive validation results.

#### 3.5.3. Controlled Observation-Quality Factors and Paired Training

The controlled operators and reference conventions below define the observation-quality factors. Let $x\in {\left[0,1\right]}^{640\times 640\times 3}$ denote a resized image. Relative GSD $r=g/g_{\mathrm{ref}}$ is simulated through anti-aliased bicubic downsampling to round(640/r) pixels per side, followed by bicubic reconstruction to 640×640.

An isotropic Gaussian PSF models the added blur. Equation (8) gives $\sigma_{q}=\sqrt{-2\log q}/\pi$, with kernel width $2\lceil 3\sigma_{q}\rceil +1$. Shot noise is applied after resampling and blur. Let *u* denote the signal entering this noise stage. For target SNR *s*, the Poisson rate is $\lambda =\bar{u}\;10^{s/10}/\bar{u^{2}}$. The output Poisson(λu)/λ is clipped to [0,1]. Here, *s* controls the expected signal-to-noise power ratio before clipping. Transformation order and random seeds remain fixed across models.

The clean training view is assigned reference labels g=1, q=0.50, and s=30 dB without adding the degradation operators. These labels set the prior to its neutral reference state. Each source image produces one clean view and one degraded view, giving a 1:1 pairing ratio. A batch contains 16 source images. Detection losses are evaluated on both views and averaged before each optimizer update.

The degraded view contains blur only with probability 0.35, Poisson noise only with probability 0.35, and both operators with probability 0.30. We draw q∼U(0.15,0.45) for blur and s∼U(10,28) dB for noise. Relative GSD remains one during training. Each metadata field is masked independently with probability $p_{\mathrm{drop}}=0.25$.

Geometric and photometric augmentation is sampled once per source image before the two views are constructed. The clean and degraded views share the same crop, resize, flip, and labels. The training seed, epoch, and image identifier determine the degradation state. Resolution changes and all operators reserved for held-out evaluation are excluded from training. Table 4 lists the resulting view-sampling protocol.

#### 3.5.4. Descriptor Correspondence and Held-Out Observation Conditions

The evaluation follows the mechanism chain

$$\mathrm{acquisition}\;\mathrm{condition}\;⟶\;\rho^{\mathrm{phy}}\;⟶\;\left(w_{3},w_{4},w_{5}\right)\;⟶\;\mathrm{detection}\;\mathrm{performance}.$$

The 27-condition grid varies known observation factors, correspondence controls alter only the image–descriptor pairing, routing probes inspect the resulting P3–P5 allocation, and held-out operators test performance outside the development family.

The factorial grid contains all 3×3×3=27 combinations of relative GSD {1,2,3}, MTF {0.50,0.30,0.15}, and SNR {30,20,10} dB. The internal visual-only baseline and PIR-SBFR are evaluated in each cell with the ten common seeds 2023–2032. The reported cell differences are means of seed-paired AP differences.

In the ten-run grid protocol, all added degradation operators are bypassed in the (1,0.50,30) reference cell, which serves as the clean anchor.

A separate mixed-degradation DIOR set tests metadata correspondence. Every image has at least one non-reference GSD, MTF, or SNR setting, ensuring that a⊙d is informative. Images and the three retained model checkpoints remain fixed across control conditions; only descriptor values and availability masks change. Results are three-checkpoint mean within-set differences from correctly paired metadata.

Nine held-out conditions use operators excluded from training, early stopping, hyperparameter selection, and model selection. The unseen PSFs comprise disk defocus with a three-pixel radius, nine-pixel motion blur with random orientation, and anisotropic Gaussian blur with $\sigma_{x}=2.5$, $\sigma_{y}=0.6$, and random orientation.

The unseen noise conditions are multiplicative speckle with σ=0.12 and stripe noise with amplitude 0.08 plus read noise with σ=0.02. Three additional conditions apply anti-aliased 1.5×, 2.5×, and 4× sampling changes. The final condition combines 2.5× sampling with an unseen PSF and unseen noise.

Each held-out value is a three-seed mean. The joint-condition record identifies the operator families but omits the sampled PSF orientation. Conversion of the non-Gaussian PSFs and non-Poisson noise to scalar *q* and *s* inputs is incompletely documented, limiting exact reconstruction of these tests.

#### 3.5.5. Controlled Implementation and Training Details

The YOLOv11n, YOLOv11s, internal-baseline, and PIR-SBFR configurations are implemented in a common PyTorch 2.8.0 detection scaffold derived from the YOLOv11n codebase. They are trained from scratch under Ubuntu 22.04 with CUDA 12.8 and share optimization, assignment, and evaluation procedures. The matched Transformer comparators use adapted official implementations, retain the architecture-specific optimizer settings stated in Section 3.5.2, and share the image views, evaluator, training duration, and selection budget defined there. Training uses one NVIDIA RTX 4090D GPU with 24 GB of memory and an AMD EPYC 9754 processor. The input size is 640×640, and training lasts 200 epochs.

For the YOLO-derived configurations, stochastic gradient descent uses a learning rate of 0.005, momentum of 0.937, weight decay of $5\times 10^{-4}$, and three warm-up epochs. Mosaic probability is 1.0 during epochs 1–180 and zero during the final 20 epochs. Every training sample receives a hue–saturation–value (HSV) perturbation. The hue offset follows U(−0.015,0.015), while saturation and value multipliers follow U(0.30,1.70) and U(0.60,1.40), respectively.

Affine scale follows U(0.50,1.50), and translation is limited to 0.10 of the image width or height. The horizontal-flip probability is 0.50. Vertical flips, rotation, shear, perspective, MixUp, and Copy-Paste are disabled. The principal endpoint, matched AI-TOD-v2, cross-host, and quality-grid comparisons use seeds 2023–2032. The component and factorial ablations retain seeds 2023–2025, as stated in their captions.

The reported configuration fixes η=1, τ=1, K=4, $\lambda_{\mathrm{scale}}=0.1$, $\lambda_{\mathrm{cons}}=0.1$, and $p_{\mathrm{drop}}=0.25$. Table 5 states their computational roles. Unit prior strength applies the specified log prior without additional rescaling, while unit temperature leaves the combined logits untempered. Each auxiliary loss is multiplied by 0.1 alongside the detection objective. Field dropout retains each descriptor in 75% of training samples while exposing the router to missing-field states.

An additional validation-only sensitivity study retrained K∈{1,2,4,8} five times per setting. Alternative-*K* settings were compared on the DIOR validation partition. Validation AP improves from 64.41±0.32 at K=1 to 65.07±0.31 at K=4, then remains 65.02±0.32 at K=8. The larger bank also increases parameters and latency. These results support K=4 as an accuracy–cost trade-off on DIOR validation.

The remaining settings follow validation-only coefficient scans. At η=0.5,1,1.5, clean validation AP is 64.80, 65.07, and 64.96; at τ=0.75,1,1.25, it is 64.96, 65.07, and 65.01. Each auxiliary coefficient is scanned over {0,0.05,0.1,0.2,0.4}, with 0.1 giving the highest clean validation AP in both scans. Increasing $\lambda_{\mathrm{cons}}$ to 0.2 raises mixed-degradation AP from 54.18 to 54.29 but lowers clean AP from 65.07 to 64.95.

Descriptor dropout is selected over {0,0.1,0.25,0.4,0.6} using the predefined validation score $S=0.1AP_{\mathrm{clean}}+0.4AP_{\mathrm{paired}}+0.3AP_{\mathrm{miss}50}+0.2AP_{\mathrm{miss}100}$. Here, paired denotes correctly paired degraded images, and miss50/miss100 denote 50%/100% field removal. The selected value 0.25 gives the highest score, 54.17, compared with 53.56 at zero dropout and 53.96 at 0.4. These coefficient summaries provide descriptive selection evidence; run-level records and uncertainty are retained for the *K* study in Table 5b.

Desktop efficiency is measured separately on an RTX 4090 using 16-bit floating-point precision (FP16), a batch size of 1, and 640×640 inputs. The input tensor is preallocated on the graphics processing unit (GPU). We perform 200 warm-up passes, followed by five runs of 1000 timed passes. CUDA is synchronized before and after each run. Latency is the median of the five run means, and frames per second (FPS) equals 1000/latency(ms).

Desktop timing covers network forward propagation only. Disk access, decoding, resizing, host-to-device transfer, non-maximum suppression (NMS), and serialization are excluded. Peak video random-access memory (VRAM) is recorded after resetting the PyTorch peak-memory statistics.

Embedded measurements use a Jetson Orin NX 16 GB in its fixed 25 W mode with fixed CPU, GPU, and memory clocks, active cooling, JetPack 6.1, TensorRT 10.3, and CUDA 12.6. One FP16 engine and one INT8 engine are exported for each model at batch size 1 and 640×640 input resolution. The INT8 numerical-quantization calibration uses 1024 training images and is unrelated to sensor-response calibration. Each model receives 500 complete-pipeline warm-up passes, followed by five sessions of 2000 frames and a 30 min thermal-stability run. Board power is sampled at 20 Hz; the table reports its mean during the 30 min run and the temperature recorded at 30 min.

The embedded end-to-end boundary includes decode and resize, descriptor loading and normalization, host-to-device transfer, network inference, NMS, and serialization. The reported p50, p95, and p99 values are medians of the corresponding session-level quantiles. The listed frame rate is the reciprocal of p50 latency and is therefore labelled p50-equivalent throughput, not independently measured sustained throughput. Exported-engine AP is evaluated on the official DIOR test partition with the same COCO evaluator, 100 maximum detections, and no test-time augmentation. Clean images receive neutral reference descriptors. The hardware record does not link exported engines to individual main-study seeds; exported AP is reported separately from the ten-run benchmark mean. Device logs report no clock-frequency reduction in the five timed sessions.

#### 3.5.6. Metrics and Statistical Analysis

Unless stated otherwise, AP denotes common objects in context (COCO)-style average precision over intersection-over-union (IoU) thresholds from 0.50 to 0.95 in increments of 0.05. We also report AP50, AP75, scale-specific AP, and scale-specific average recall. Precision, recall, and the F1 score are included for continuity across the internal controlled configurations. AI-TOD-v2 uses its published very-tiny, tiny, small, and medium intervals.

The principal DIOR and AI-TOD-v2 comparisons use ten independently trained baseline–PIR pairs with common seeds 2023–2032. For each dataset, the primary estimate is the mean paired AP difference. We report its sample SD and two-sided 95% paired *t*-interval with nine degrees of freedom. All ten differences are positive on both datasets; the corresponding two-sided exact sign-test value is p=0.00195.

These analyses quantify training-run variation within a fixed implementation and data protocol. Absolute metric changes are reported in percentage points. Component, factorial, and held-out-operator experiments retain three paired seeds unless their captions state otherwise.

The scale-supervision/P5-bypass experiment uses a paired 2×2 design with seed as a common block. For metric *Y*, the interaction within each seed is

(27) $$\Delta_{\mathrm{int}}=Y_{11}-Y_{10}-Y_{01}+Y_{00},$$

where the subscripts indicate the presence of scale supervision and the P5 bypass. Cell values and paired interactions are summarized by their three-seed means and sample SDs. Factorial effects are interpreted descriptively.

## 4. Results

Results are organized by evidential role. Public benchmarks first establish the endpoint pattern previewed in Figure 1 under a shared implementation. Controlled alternatives then separate content-only fusion, unrestricted descriptor injection, and the ordered reliability interface. Descriptor interventions and routing probes connect acquisition conditions to P3–P5 allocation, while controlled observation-quality tests and held-out degradations relate these responses to detection performance.

### 4.1. Detection Performance Across Optical Imaging Regimes

#### 4.1.1. DIOR: Improving Fine Evidence Without Sacrificing Broad Structure

DIOR first assesses endpoint performance across a broad object-scale distribution. Table 6 reports ten independently trained, seed-paired runs. PIR-SBFR reaches 65.52±0.40 AP, compared with 62.98±0.32 for the internal visual-only baseline. The paired AP gain is 2.54±0.32 percentage points (pp), and its 95% interval is [2.31, 2.77] pp.

The largest reported scale-specific difference occurs for small objects. ${\mathrm{AP}}_{S}$ rises by 3.52 pp, while ${\mathrm{AP}}_{L}$ rises by 1.62 pp. AP75 improves by 3.07 pp. At the validation-frozen confidence threshold, precision, recall, and F1 increase from 90.10, 80.75, and 85.17% to 90.70, 82.60, and 86.46%, respectively.

The visual-demand/P5-preservation and no-consistency controls retain the original three-seed protocol and are therefore reported with the component experiments below. The cross-host analysis in Section 4.3 separates the interface gain from the stronger internal architecture.

#### 4.1.2. AI-TOD-v2: Matched Tiny-Object Comparison

AI-TOD-v2 tests the formulation near the tiny-object limit. Table 7 compares all methods under the matched protocol in Section 3.5.2. Across ten paired runs, PIR-SBFR improves AP from 29.58±0.27 to 32.10±0.41, a mean difference of 2.52±0.38 pp with a 95% interval of [2.25, 2.79] pp.

PIR-SBFR also exceeds the controlled local reproductions of DQ-DETR, DINO-D3Q, and Dome-DETR-M by 1.674, 0.804, and 0.510 pp AP, respectively. The interval for the smallest difference, against Dome-DETR-M, is [0.382, 0.638] pp.

The strongest gains occur in strict localization and the smallest scale intervals. PIR-SBFR obtains the highest AP75, ${\mathrm{AP}}_{\mathrm{VT}}$, and ${\mathrm{AR}}_{\mathrm{VT}}$ in the matched table. Dome-DETR-M remains higher for medium-object AP, at 47.42% versus 46.17%.

### 4.2. Controlled Alternatives to Observation-Constrained Allocation

Table 8 compares content-only fusion, degradation exposure, freely learned metadata conditioning, and the ordered analytic route. All configurations use the same images, training duration, and evaluator. The held-out column averages the nine operators excluded from training. Metadata shuffling is measured on the separate mixed-degradation correspondence set.

Degradation augmentation improves held-out AP from 46.95 to 48.02. ASFF reaches 48.47 using image content alone. Metadata-FiLM reaches 49.71, while the analytic route reaches 50.36. PIR-SBFR combines scene demand and ordered reliability and reaches 51.32. The complete demand–reliability interface outperforms each tested alternative.

Domain generalization reduces sensitivity to unseen domains by learning invariant or diversified visual representations [29]. It normally does not use an image-synchronized acquisition descriptor at inference. PIR-SBFR instead permits the supplied observation state to alter the allocation for each image. The two goals are complementary rather than interchangeable.

### 4.3. Cross-Host Validation

The internal visual architecture already exceeds stock YOLOv11n by 5.67 pp AP. That improvement comes from the shared internal detector. Table 9 therefore tests the routing interface on three compatible hosts. PIR-SBFR improves YOLOv11n, YOLOv11s, and the internal architecture by 2.25, 2.09, and 2.54 pp, respectively.

Every paired interval excludes zero, and the smallest model remains the most sensitive to the added parameters. The gains across all three hosts show that the interface also benefits detectors without DRFB or FACH.

### 4.4. Exploratory Channel-Gating Control

Table 10 reports the validation-only channel-gating control defined in Section 3.5.2. The original PIR-SBFR configuration reaches 65.07±0.31 AP across five independent trainings. Adding a content-only channel gate changes the mean by +0.07 pp, while descriptor-conditioned channel gating changes it by +0.10 pp. Both increments are small relative to the marginal run SDs. Paired-difference uncertainty was not retained, so these changes are descriptive only.

Given the small mean gains, the present model retains its original scale-routing design.

### 4.5. Controlled Analysis of the Sensor-to-Inference Interface

#### 4.5.1. Separating Observation Reliability, Scene Demand, and Prediction Consistency

Table 11 contrasts information paths under the shared extractor and prediction mapping. A1 tests degradation exposure without descriptor routing. A2 tests scene-demand supervision and direct P5 preservation without an acquisition prior. A3–A5 activate analytic observation constraints, whereas A6–A7 use unconstrained descriptor injection. A8 reconciles the analytic prior with visual demand, and A9 additionally applies cross-view consistency.

The MTF/PSF-only and SNR-only analytic routes reach 64.11 and 63.93 AP. The combined analytic route reaches 64.87 AP and 46.77 ${\mathrm{AP}}_{S}$, with GSD fixed at its reference throughout paired training and clean evaluation. GSD response is evaluated through the controlled grid, routing probes, and held-out sampling conditions.

The combined analytic prior exceeds direct concatenation by 0.89 pp and Metadata-FiLM by 0.47 pp. A8 is 0.30 pp higher than the combined analytic route, and A9 is 0.35 pp higher than A8. Across the reported interface designs, ${\mathrm{AP}}_{S}$ ranges from 44.08 to 47.61. The MTF/PSF-only route exceeds the SNR-only route by 0.18 pp.

No single tested information path matches the full reconciled interface. The focused experiment below separately examines scene-demand supervision and direct P5 preservation.

#### 4.5.2. Scene-Demand Supervision and Coarse-Evidence Preservation

Table 12 uses A1 as the common baseline in a paired 2×2 experiment. Every cell retains degradation augmentation, seeds 2023–2025, and the same training protocol. Only scale supervision and the P5 structural bypass vary. The design separates demand estimation from coarse-evidence preservation.

Scale supervision alone raises AP by 0.20 pp and ${\mathrm{AP}}_{S}$ by 0.45 pp, while changing ${\mathrm{AP}}_{L}$ by −0.11 pp. The P5 bypass alone adds 0.11 pp AP, 0.13 pp ${\mathrm{AP}}_{S}$, and 0.09 pp ${\mathrm{AP}}_{L}$. Relative to B0, the joint configuration adds 0.32 pp AP, 0.56 pp ${\mathrm{AP}}_{S}$, and 0.07 pp ${\mathrm{AP}}_{L}$.

After averaging over the other factor, scale supervision has marginal effects of +0.21 pp AP, +0.44 pp ${\mathrm{AP}}_{S}$, and −0.07 pp ${\mathrm{AP}}_{L}$. The corresponding bypass effects are +0.12, +0.12, and +0.14 pp.

The paired interactions are +0.01 pp for AP, −0.02 pp for ${\mathrm{AP}}_{S}$, and +0.09 pp for ${\mathrm{AP}}_{L}$. AP is nearly additive at the observed precision. The positive ${\mathrm{AP}}_{L}$ interaction is consistent with the intended compensating role of the bypass.

**Figure 6:**
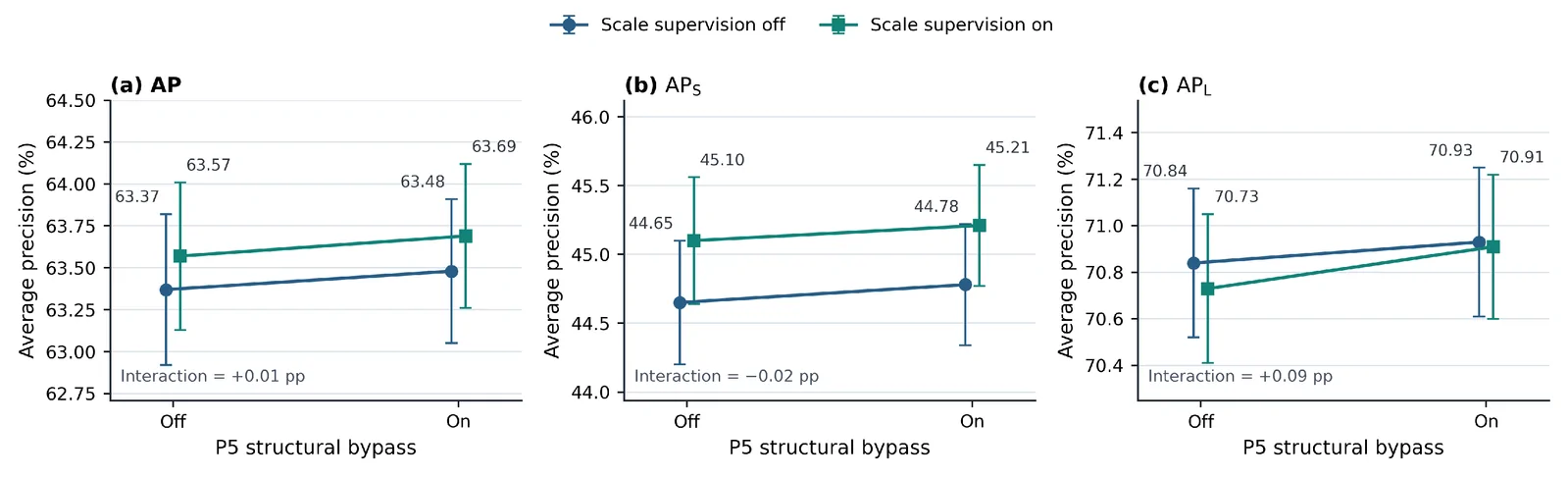
Factorial interaction of scale supervision and the P5 structural bypass on DIOR. Points show three-seed means, and error bars show sample SD. Each panel reports the paired interaction from Equation (27). Panels use metric-specific vertical ranges.

Figure 6 visualizes the same cells. The nearly parallel AP and ${\mathrm{AP}}_{S}$ trajectories agree with their small interactions. For ${\mathrm{AP}}_{L}$, the converging trajectories are consistent with the intended compensating role of direct P5 preservation.

### 4.6. Evidence Chain from Acquisition Conditions to P3–P5 Allocation

#### 4.6.1. Descriptor Correspondence and Missing-Field Controls

If acquisition descriptors influence inference through the ordered prior, performance should depend on their correspondence with the observed image. Table 13 tests this implication on the mixed-degradation set while holding images and checkpoints fixed. Correct pairing is the zero reference. Increasing descriptor error produces a graded difference: AP changes by −0.28, −0.76, and −1.64 pp as multiplicative error increases from ±10% to ±50%.

Masking 25%, 50%, and 100% of the fields changes AP by −0.61, −1.15, and −1.80 pp, respectively. Complete masking leaves the visual allocation path operational, but AP is 1.80 pp below the correctly paired condition. Replacing every descriptor with the training-set mean gives a 1.67 pp difference.

Cross-image shuffling preserves the marginal descriptor distribution while destroying sample correspondence. It produces the largest AP loss, −2.38 pp, and lowers ${\mathrm{AP}}_{S}$ by 3.24 pp. This pattern is inconsistent with an explanation based only on a constant metadata offset. It supports sample-specific descriptor use on the mixed-degradation set.

#### 4.6.2. Routing Response to Observation-Quality Descriptors

**Figure 7:**
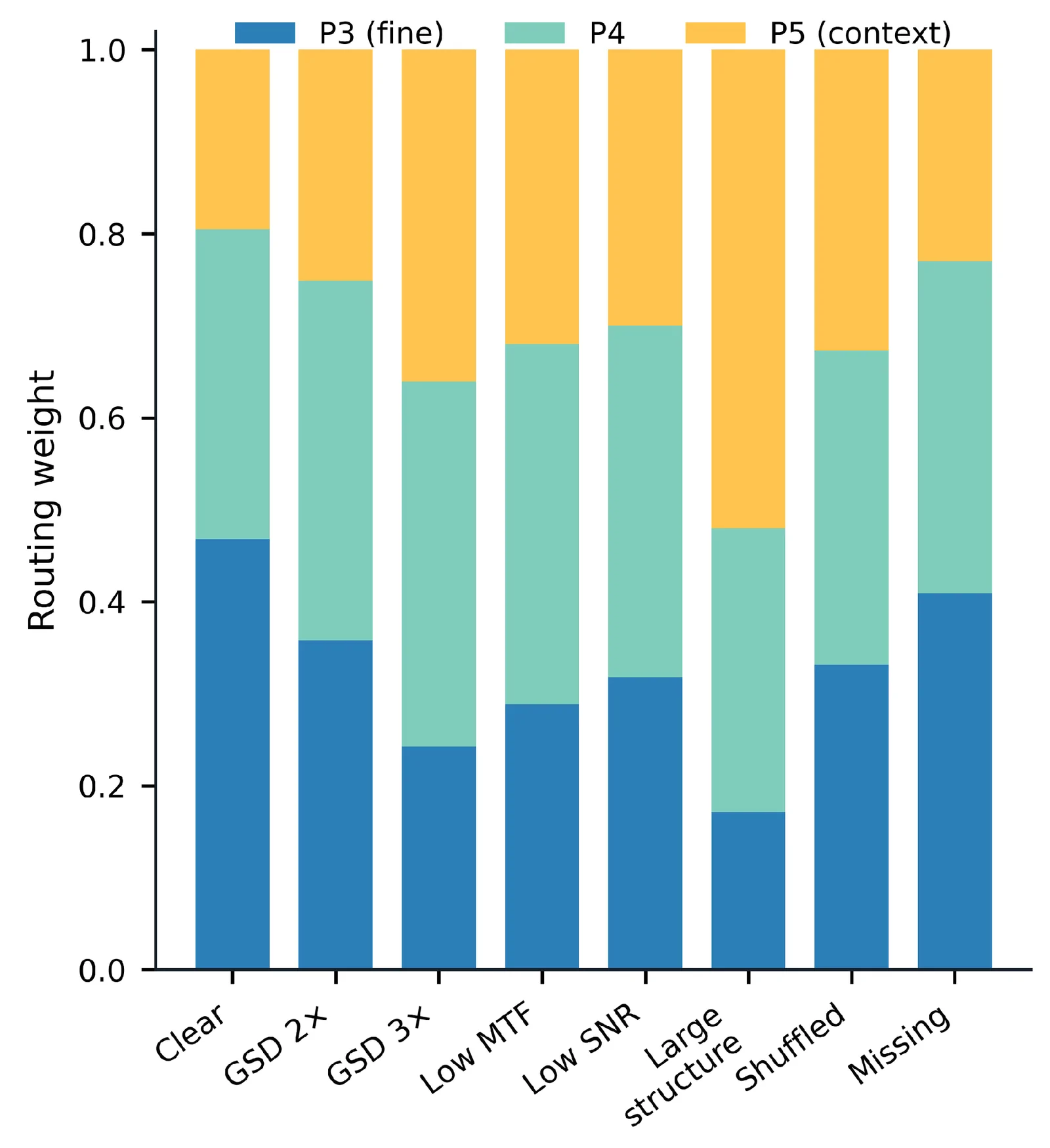
Descriptive aggregate routing weights under controlled observation-quality, scene-content, shuffled-metadata, and missing-metadata conditions. Each P3–P5 stack sums to one. These probes are not independent training-run endpoints.

Figure 7 connects controlled acquisition conditions to the resulting P3–P5 allocation. The ordered prior predicts lower relative support for P3 under coarser sampling. Correspondingly, the P3 weight changes from 0.468 under clear conditions to 0.242 at relative GSD 3×, while the P5 weight changes from 0.195 to 0.361. Lower MTF and SNR produce the same directional shift from fine to contextual evidence.

Scene content can oppose the acquisition-driven shift. In the clear large-structure probe, visual demand raises the P5 weight to 0.520. Shuffled descriptors produce nearly uniform weights and the largest earth-mover discrepancy, 0.0909, from the annotated scale distribution. When all descriptors are masked, the visual path still supplies a valid allocation.

These trajectories follow the directions specified by the two inputs: object composition is associated with visual demand, whereas acquisition descriptors alter the analytic constraint. The correspondence controls and condition-specific performance provide complementary evidence for this allocation pattern.

### 4.7. Detection Performance Along the Observation-Quality Grid

**Figure 8:**
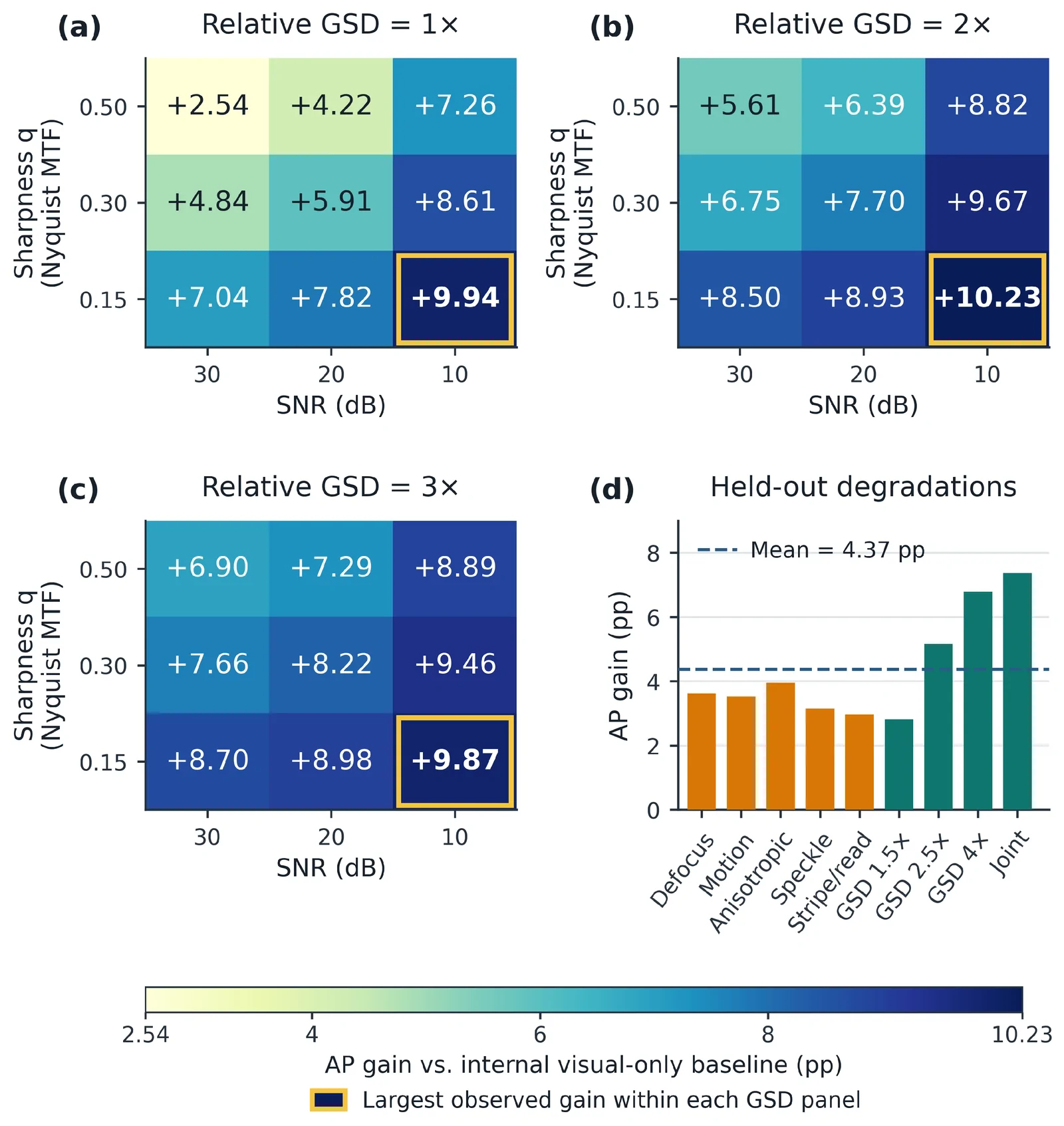
Performance differences under controlled observation-quality changes. (**a**–**c**) Ten-seed mean AP gain of PIR-SBFR over the seed-paired internal visual-only baseline for the complete GSD–MTF–SNR grid, using a shared percentage-point color scale. Outlined cells mark the largest observed gain in each GSD panel. The global maximum is 10.23 pp in panel (**b**); the outlines are descriptive and do not denote statistical significance. (**d**) Three-seed mean gains for nine degradation types held out from training and model selection. Orange bars denote non-GSD operators; teal bars denote GSD changes and the joint condition. The dashed line marks their mean gain of 4.37 pp.

The quality grid evaluates the performance difference as fine-scale evidence degrades. Figure 8a–c reports this comparison after the descriptor-to-prior and prior-to-routing responses have been examined above. PIR-SBFR exceeds the internal visual-only baseline in all 27 reported GSD–MTF–SNR cells. Gains range from 2.54 pp in the reference-labelled cell to 10.23 pp at relative GSD 2×, nominal MTF 0.15, and nominal SNR 10 dB. The grid-wide mean is 7.66 pp.

At MTF 0.30 and SNR 20 dB, the gain rises from 5.91 pp at relative GSD 1× to 7.70 pp at 2× and 8.22 pp at 3×. This trend differs from a constant clean-set offset. Along the fixed optical and noise slice, the advantage increases with the sampling constraint.

Table 14 and Figure 8d examine transfer beyond the training operators. All nine held-out degradations yield positive differences from 2.82 to 7.37 pp. Mean AP rises from 46.95 to 51.32, and mean ${\mathrm{AP}}_{S}$ improves by 6.33 pp. Mean retention relative to clean performance increases from 74.55% to 78.33%.

Unseen 4× sampling yields a 6.79 pp gain. The combined unseen condition is the lowest-performing tested case for both detectors. PIR-SBFR raises AP from 29.60 to 36.97 and raises the lowest AP observed in the tested conditions.

### 4.8. Training-Run Stability and Class-Wise Pattern

The AP difference is positive in every one of the ten paired runs on each dataset. On DIOR, the run-level differences range from 1.88 to 2.88 pp. On AI-TOD-v2, they range from 2.00 to 3.10 pp. Table 15 reports the paired effect estimates. These replications quantify training-run stability under the fixed protocol.

The paired *t*-intervals exclude zero, and the exact two-sided sign-test result is p=0.00195 for each dataset. AP is the prespecified endpoint in this analysis.

**Figure 9:**
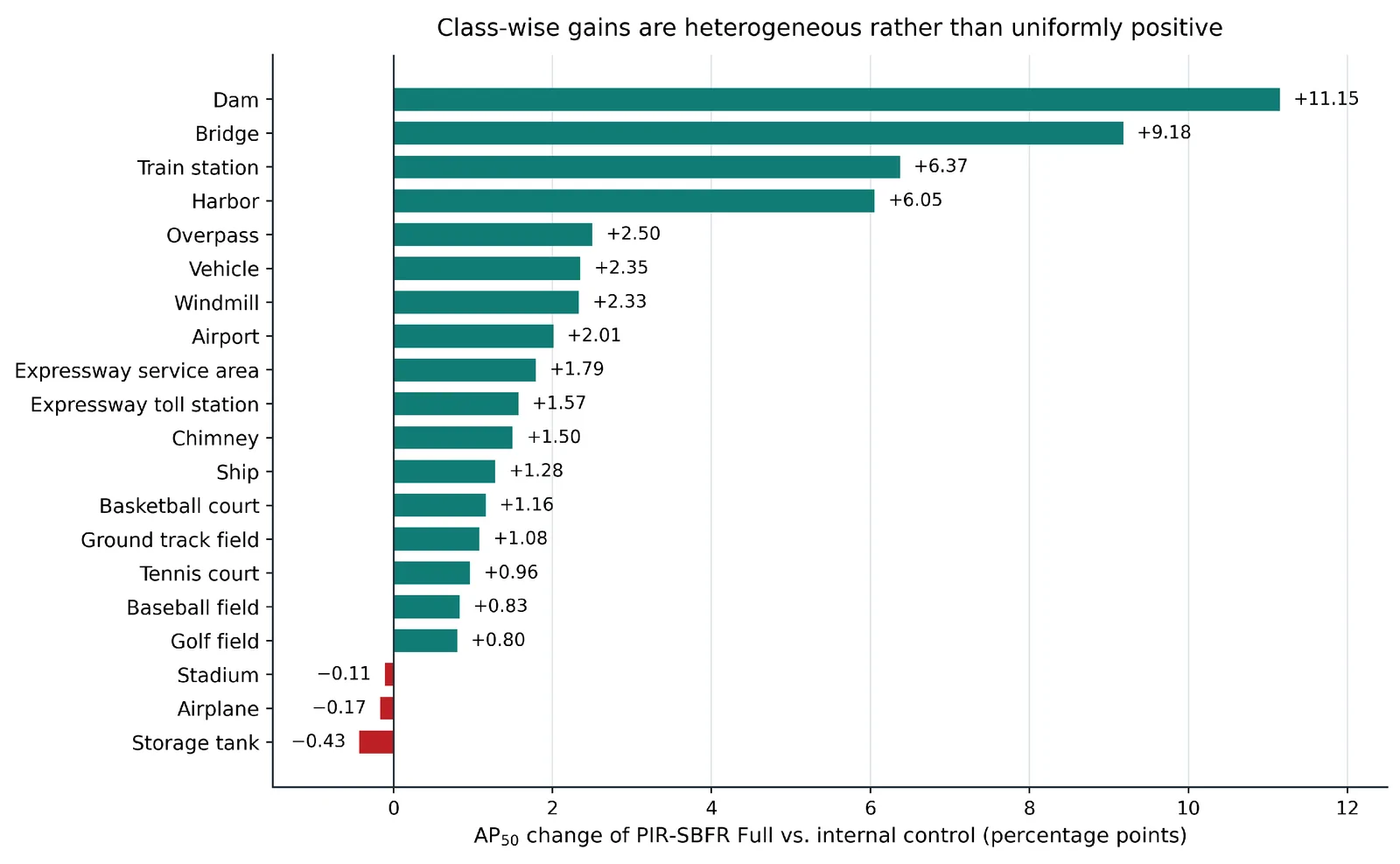
Retained three-seed mean class-wise AP50 change of PIR-SBFR Full relative to the internal visual-only baseline on DIOR. Green bars indicate improvements and red bars indicate declines. Classes are ordered by the measured change; no category is omitted. This class-wise analysis is separate from the ten-run primary AP endpoint.

Figure 9 shows positive AP50 changes for 17 of the 20 DIOR categories. Airplane, stadium, and storage tank decline by 0.17, 0.11, and 0.43 pp, respectively. Dam and bridge show the largest gains, at 11.15 and 9.18 pp. Train station and harbor improve by 6.37 and 6.05 pp.

Storage tank also remains below its YOLOv11n result. Spatial allocation offers a direction for examining category and regional differences further.

### 4.9. Desktop and Embedded Computational Cost

Table 16 quantifies the computational cost of the routing interface. PIR-SBFR adds 0.314 M parameters and 0.39 GFLOPs over the internal visual-only baseline, corresponding to 8.65% and 4.63%. On the RTX 4090, forward latency rises from 3.079 to 3.313 ms, while forward throughput decreases from 324.8 to 301.8 FPS. The corresponding ten-run gains are 2.54 pp AP and 3.52 pp ${\mathrm{AP}}_{S}$.

The Jetson measurements include the complete boundary specified in Section 3.5.5. The PIR-SBFR FP16 engine achieves 65.49% AP and has p50/p95/p99 latencies of 19.55/20.47/21.63 ms. The FP16 p50 increment over the internal baseline is 1.19 ms. The INT8 engine reaches 65.13% AP at 16.03 ms p50. Device logs report no clock-frequency reduction in the five timed sessions.

The p50-equivalent rates are 51.15 FPS for FP16 and 62.38 FPS for INT8. They are reciprocals of median latency, not independent sustained-throughput measurements. Power rises from 17.6 to 18.2 W in FP16 and from 16.2 to 16.9 W in INT8. Consistency loss is training-only and does not alter the inference graph.

## 5. Discussion

### 5.1. Feature-Map Scale and Sensor-Supported Evidence

Pyramid labels describe network stride; they do not measure the spatial evidence preserved by acquisition. P3 remains the finest tensor even when sampling, optical transfer, or noise has removed much of its useful detail. An optical perception system must determine both which scale matches an object and which scale still carries credible evidence.

The demand–reliability mismatch reframes multiscale routing from selecting among tensor resolutions to allocating evidence under acquisition constraints. On DIOR, PIR-SBFR improves AP by 2.54 percentage points (pp), including gains of 3.52 pp for small objects and 1.62 pp for large objects. On AI-TOD-v2, AP improves by 2.52 pp. Matched local reproductions place the largest comparative advantage in strict localization and very-tiny-object detection.

Controlled alternatives, internal probes, and descriptor interventions explain the mechanism associated with these benchmark improvements.

### 5.2. Evidence for Reliability-Aware Multiscale Perception

The same-code comparison makes a pure model-capacity explanation less likely. Degradation augmentation, ASFF, Metadata-FiLM, and the analytic-only route remain below PIR-SBFR on both clean and held-out observations. The matched AI-TOD-v2 comparison also places PIR-SBFR above three contemporary local reproductions in overall AP.

Cross-host results address the stronger internal detector directly. Adding the same interface improves AP by 2.25 pp on YOLOv11n, 2.09 pp on YOLOv11s, and 2.54 pp on the internal architecture. These paired comparisons separate the routing gain from the shared detector architecture.

The paired 2×2 ablation addresses a second ambiguity inside the visual-demand/P5-preservation control. Scale supervision has a +0.44 pp marginal effect on ${\mathrm{AP}}_{S}$, while the P5 bypass has a +0.14 pp marginal effect on ${\mathrm{AP}}_{L}$. Their AP interaction is +0.01 pp. The ${\mathrm{AP}}_{L}$ pattern is consistent with the intended compensating role of the bypass.

Correspondence controls examine whether a constant metadata offset could account for the result. Replacing paired descriptors with the training-set mean reduces AP by 1.67 pp. Complete masking produces a 1.80 pp reduction, and cross-image shuffling causes a 2.38 pp loss. Checkpoints and degraded images remain fixed. The measured difference depends on descriptor alignment within the mixed-degradation set.

Internal probes exhibit the predicted response pattern. Coarser GSD, lower MTF, and lower SNR shift allocation away from P3 and toward contextual levels. Under good image quality, large-structure scenes increase P5 through the visual path. Acquisition quality and object composition move different parts of the router. The weight trajectories match the variables assigned to each branch.

Proposition 1 separates PIR-SBFR from an unrestricted attention rule at the mechanism level. For fixed visual demand, the analytic branch guarantees the direction of every available degradation coordinate in pairwise P3–P5 allocation odds.

The controlled observation-quality tests connect the internal response direction to measured performance. PIR-SBFR improves all 27 reported GSD–MTF–SNR cells, with a mean gain of 7.66 pp. Along the reported fixed-sharpness, fixed-noise slice, the advantage increases as sampling becomes coarser. Nine held-out degradations yield a mean improvement of 4.37 pp. The joint stress condition gains 7.37 pp, although absolute performance remains low at 36.97 AP.

Across these tests, acquisition-reliability interventions change both routing weights and detection performance in the predicted direction.

### 5.3. A Calibration-Free Sensor-to-Inference Routing Interface

PIR-SBFR treats acquisition descriptors as low-bandwidth context accompanying each image. It converts available descriptors into an ordered constraint and reconciles that constraint with visually estimated scene demand. For fixed visual demand, this construction gives sign-controlled pairwise scale-allocation odds.

The interface operates downstream of the optical sensor and supports variable observation quality through compatible acquisition records. Availability masks handle incomplete records, with complete masking returning the detector to visual-only routing.

### 5.4. Scale Allocation and Inter-Channel Interaction

PIR-SBFR assigns one scalar weight to each pyramid level for an entire image. The shared FACH mapping already uses global channel context and input-dependent transformations, but these operations receive no acquisition descriptors. Their role is content-based representation refinement. Holding this mapping fixed isolates the tested scale-routing intervention.

The context-conditioned reconstruction in DirectNet and the spectral priors in SPG-OD suggest complementary ways to model dependencies within a representation [30,31]. Their hyperspectral setting includes wavelength-indexed information that is absent from the evaluated RGB inputs. In an additional five-run DIOR validation check, a content-only channel gate adds 0.07 pp AP and a descriptor-conditioned gate adds 0.10 pp. These changes are small relative to the marginal run SDs of 0.31–0.33 pp, and paired-difference uncertainty was not retained.

The small descriptive gains favor retaining the current scale-routing design. Future channel-aware models could examine how acquisition quality affects different feature channels.

### 5.5. Prospective Extension to Spatial Routing

One weight per pyramid level cannot accommodate a sharp region and a blurred region independently. A spatial extension could estimate local demand on a common image-coordinate grid and normalize the three allocation weights at each location. Each resulting weight map would be resampled to its pyramid level before feature reweighting. The unweighted P5 bypass could remain unchanged. ASFF and UAV-DETR provide relevant precedents for spatially adaptive fusion and cross-level alignment [12,24].

Spatially resolved reliability would require local descriptor maps and availability masks in addition to local visual demand. Local GSD might follow a geometric projection; local blur and noise would require validated estimates with specified spatial support. Unavailable local fields should retain the neutral state.

Training would require aligned local-scale targets, a rule for regions without annotated objects, and checks for artifacts from resampling the weight maps. Dense allocation also adds intermediate tensors and memory traffic, so its accuracy and efficiency would need joint evaluation. This proposed extension remains to be implemented and evaluated through retraining and regional-quality tests.

### 5.6. Obtaining Descriptors in an Aerial Imaging System

The benchmark supplies descriptor values from known transformations. Deployment would instead require a descriptor provider upstream of the router, with values linked to the corresponding image or acquisition interval. For approximately nadir viewing over locally planar terrain, GSD can be derived from camera geometry, pixel pitch, and height above ground. Oblique views or varying terrain require a geometric model or georeferencing product rather than a height-only approximation.

A sharpness descriptor could come from a documented PSF or spatial-frequency response at the relevant optics and processing settings [18,20]. Such a nominal value would not necessarily capture frame-dependent motion blur. A non-isotropic response also requires a documented directional reduction before it can supply one scalar *q*. An image-based estimate would require separate validation and conversion to the Nyquist-MTF convention used here. SNR could be supplied by a compatible signal/noise estimate using exposure and sensor-noise characterization [19]; ISO gain alone is not an SNR measurement.

Descriptor definitions, spatial support, units, and reference settings must be compatible with training. For images without acquisition records, descriptor fields require a validated image-based estimator or are marked unavailable. If a field is unavailable, stale, or unsupported by a validated conversion, its availability flag should be zero. The router then omits the unsupported term. Descriptor accuracy and synchronization are key considerations for integrating these providers.

### 5.7. Limitations and Future Directions

The present experiments use public imagery and operator-defined descriptors. Native-sensor variation and cross-camera transfer remain untested. Evaluation with synchronized, validated descriptors from multiple cameras would establish how the routing prior responds to physical acquisition changes.

The method also uses one allocation per image. Spatial routing could address non-uniform image quality, while channel-aware designs could model acquisition-dependent feature interactions. The three category-level declines reported in Section 4 motivate closer analysis of object appearance and regional quality. Wider validation could further examine the fixed routing coefficients and transfer of the selected *K*.

### 5.8. Embedded Deployment Evidence

The Jetson experiment extends the resource analysis beyond arithmetic counts and desktop forward timing. The exported PIR-SBFR engine achieves 65.49% AP in FP16 at 19.55 ms median end-to-end latency. Its INT8 engine achieves 65.13% AP at 16.03 ms. The complete boundary includes descriptor loading, preprocessing, transfers, inference, NMS, and serialization.

Device logs report no clock-frequency reduction in the five timed sessions under the fixed 25 W mode. The 18.2 W FP16 mean and 2.47 GB peak memory remain within the tested module’s operating configuration. The median latency corresponds to a p50-equivalent rate of 51.15 FPS.

These measurements demonstrate deployment feasibility on the tested Jetson Orin NX. Further platform evaluations can examine engine conversion, numerical precision, memory traffic, and descriptor provision.

## 6. Conclusions

This study evaluated a calibration-free sensor-to-inference routing interface that carries observation-quality descriptors into multiscale visual inference. PIR-SBFR separates scene-scale demand from an image-formation-informed reliability prior over P3–P5 and reconciles the two signals in logit space. For fixed visual demand, the analytic component prescribes the direction of each available degradation coordinate in pairwise scale-allocation odds. The analytic routing rule requires no fitted sensor-specific response.

Across ten paired runs, the complete PIR-SBFR configuration improves AP by 2.54 pp on DIOR and by 2.52 pp on the study-specific AI-TOD-v2 split. The corresponding 95% paired intervals are [2.31, 2.77] and [2.25, 2.79] pp. Three compatible host detectors gain 2.09–2.54 pp, separating the interface effect from the stronger internal architecture.

Under the matched AI-TOD-v2 protocol, PIR-SBFR exceeds three contemporary tiny-object detector reproductions in overall AP. Descriptor-correspondence controls, routing probes, the 27-condition grid, and nine held-out degradations support condition-dependent allocation in the evaluated setting. K sensitivity places the four-transformation bank near the observed validation plateau.

PIR-SBFR adds 4.63% GFLOPs and 7.60% RTX FP16 forward latency over the internal baseline. On a Jetson Orin NX, its exported FP16 engine achieves 65.49% AP at 19.55 ms median end-to-end latency.

Future work will extend the evaluation to synchronized multi-camera descriptors, spatially resolved routing, and additional embedded platforms. 

## Figures and Tables

**Table 1:** Comparison of the cited multiscale and acquisition-conditioned formulations. Panel (**a**) compares routing information; panel (**b**) compares descriptor handling and evaluation. Correspondence controls describe experimental designs rather than architectural capabilities. FPN: Feature Pyramid Network; BiFPN: Bidirectional Feature Pyramid Network; ASFF: Adaptively Spatial Feature Fusion.

**(a) Sources of routing information**			
**Method Family**	**Scene-Scale Demand**	**Acquisition Reliability**	
FPN/BiFPN [10,11]	Indirect, via feature fusion	Not explicit	
ASFF/content-driven attention [12]	Content-derived	Not explicit	
Direct metadata conditioning [15,16]	Indirect	Freely learned	
PIR-SBFR	Scale-supervised	Analytic prior	
**(b) Robustness and mechanism controls**			
**Method Family**	**Missing-Field Handling**	**Directional P3–P5 Constraint**	**Correspondence control**
FPN/BiFPN [10,11]	No descriptor input	No explicit acquisition constraint	No descriptor input
ASFF/content-driven attention [12]	No descriptor input	No explicit acquisition constraint	No descriptor input
Direct metadata conditioning [15,16]	Model-specific	Not guaranteed	Not explicit in cited methods
PIR-SBFR	Mask and neutral fallback	Guaranteed for fixed demand	Evaluated

**Table 2:** Provenance and handling of the acquisition descriptors used by PIR-SBFR.

Descriptor	Observation Meaning	Public-Benchmark Handling
Relative GSD	Projected sampling footprint	Controlled evaluation factor; fixed at 1 during paired training
Nominal Nyquist MTF	Spatial-detail transfer	Ideal Gaussian-PSF parameter; reference label for an unmodified view
Nominal SNR	Noise stability	Poisson-noise target before clipping; reference label for an unmodified view
Availability mask	Descriptor presence	Controlled field dropout

**Table 3:** Public-dataset image partitions used by every method run in this study. DIOR follows its official partitioning; AI-TOD-v2 follows the fixed study partition described in the text. Counts refer to unique image identifiers.

Dataset	Partition	Images
DIOR	Training	5862
DIOR	Validation	5863
DIOR	Test	11,738
AI-TOD-v2	Training	11,214
AI-TOD-v2	Validation	5607
AI-TOD-v2	Test	11,215

**Table 4:** Sampling of the paired training views. A dash indicates that the corresponding degradation operator is not applied.

View or Mode	Probability	Relative GSD	MTF Parameter	SNR Parameter
Clean view	1.00	1	0.50 reference	30 dB reference
Blur-only degraded view	0.35	1	U(0.15,0.45)	–
Noise-only degraded view	0.35	1	–	U(10,28) dB
Joint degraded view	0.30	1	U(0.15,0.45)	U(10,28) dB

**Table 5:** Routing settings and validation-only sensitivity analysis. Panel (**a**) states the selected values and roles. Panel (**b**) summarizes five independent DIOR validation trainings for each visual-router bank size. AP and scale-specific metrics are five-run means; parameters, GFLOPs, and latency are single standardized profiles.

**(a) Selected routing settings**						
**Setting**	**Value**	**Role and Interpretation**				
*K*	4	Number of visual transformations; each produces three scale logits.				
η	1	Strength of the analytic log prior; zero removes its contribution.				
τ	1	Softmax temperature; lower values sharpen allocation at fixed logits.				
$\lambda_{\mathrm{scale}}$	0.1	Coefficient of the scene-scale supervision loss.				
$\lambda_{\mathrm{cons}}$	0.1	Coefficient of paired-view prediction consistency.				
$p_{\mathrm{drop}}$	0.25	Independent masking probability for each descriptor during training.				
**(b) Visual-router bank size** ***K***						
***K***	**Validation AP**	${\mathrm{AP}}_{S}$	${\mathrm{AP}}_{L}$	**Params (M)**	**GFLOPs**	**RTX Latency (ms)**
1	64.41±0.32	46.43	71.68	3.910	8.79	3.287
2	64.83±0.29	46.74	71.81	3.921	8.80	3.296
4	65.07±0.31	**47.11**	**71.93**	3.942	8.82	3.313
8	65.02±0.32	47.08	71.91	3.984	8.86	3.347

Bold metric values identify the highest validation AP, small-object AP, and large-object AP in panel (**b**).

**Table 6:** Ten-run paired comparison on DIOR. Values are percentages and are shown as mean ± sample SD over seeds 2023–2032. Best values are bold.

Model	AP50	AP75	AP	${\mathrm{AP}}_{S}$	${\mathrm{AP}}_{L}$
Internal visual-only baseline	82.30±0.34	67.62±0.39	62.98±0.32	44.06±0.48	70.55±0.34
**PIR-SBFR Full**	84.91±0.31	70.69±0.35	65.52±0.40	47.58±0.43	72.17±0.31

**Table 7:** Ten-run matched local comparison on the study-specific AI-TOD-v2 split. Every method uses the same image identifiers, 640×640 inputs, 200 epochs, view count, and evaluator. AP is mean ± sample SD; other detection metrics are ten-run means. VT denotes very tiny.

**(a) Aggregate detection metrics**					
**Method**	**AP50**	**AP75**	**AP**	**AR**	
Internal visual-only baseline	53.68	28.30	29.576±0.272	41.10	
DQ-DETR [25]	55.88	29.17	30.422±0.259	42.72	
DINO-D3Q [26]	56.34	30.22	31.292±0.271	43.36	
Dome-DETR-M [27]	56.61	30.69	31.586±0.272	44.02	
**PIR-SBFR Full**	**56.89**	**31.18**	32.096±0.410	**44.54**	
**(b) Scale-specific performance**					
**Method**	${\mathrm{AP}}_{\mathrm{VT}}$	${\mathrm{AP}}_{T}$	${\mathrm{AP}}_{S}$	${\mathrm{AP}}_{M}$	${\mathrm{AR}}_{\mathrm{VT}}$
Internal visual-only baseline	14.58	28.38	39.68	45.00	23.90
DQ-DETR [25]	15.83	29.42	40.83	46.52	25.31
DINO-D3Q [26]	16.36	30.11	41.36	47.18	26.22
Dome-DETR-M [27]	16.76	30.57	41.91	**47.42**	26.74
**PIR-SBFR Full**	**17.16**	**30.99**	**42.27**	46.17	**27.39**

Bold metric values indicate the highest value in each column; the proposed method name is also bold.

**Table 8:** Controlled alternatives on DIOR. Values are retained three-seed means. Clean and held-out values are percentages. Shuffle effects are percentage-point changes on the separate mixed-degradation correspondence set. N/A denotes a method without acquisition descriptors.

Method	Clean AP	Held-Out AP	Held-Out ${\mathrm{AP}}_{S}$	Shuffle ΔAP	Routing Information
Internal visual-only baseline	62.98	46.95	28.39	N/A	Scene content
Deg.-augmented baseline	63.37	48.02	29.54	N/A	Scene content
ASFF [12]	62.28	48.47	30.03	N/A	Spatial content weights
Metadata-FiLM [16]	64.40	49.71	32.08	−1.12	Freely learned metadata conditioning
Analytic reliability route	64.87	50.36	33.01	−1.86	Ordered observation prior
**PIR-SBFR Full**	**65.52**	**51.32**	**34.72**	−2.38	Scene demand + ordered reliability

Bold entries identify the full PIR-SBFR configuration; bold does not indicate that a larger shuffle loss is preferable.

**Table 9:** Ten-run cross-host comparison on DIOR. AP and paired gains are percentages and percentage points, respectively. Parameter and GFLOP entries list baseline/PIR-SBFR values. Intervals are based on seed-paired AP differences.

Host	Base/PIR AP	Gain	95% Interval	Params (M)	GFLOPs
YOLOv11n	57.31/59.56	+2.25	[1.86, 2.64]	2.584/2.898	6.47/6.85
YOLOv11s	61.84/63.93	+2.09	[1.73, 2.45]	9.460/9.774	21.50/22.06
Internal visual architecture	62.98/65.52	**+2.54**	[2.31, 2.77]	3.628/3.942	8.43/8.82

The bold gain is the largest paired AP improvement across the three hosts.

**Table 10:** Exploratory five-run channel-gating control on the DIOR validation set. AP is mean ± sample SD; scale-specific metrics are five-run means. Changes are relative to the original PIR-SBFR configuration and are expressed in percentage points.

Configuration	AP	${\mathrm{AP}}_{S}$	${\mathrm{AP}}_{L}$	Params (M)	GFLOPs	ΔAP
Original PIR-SBFR	65.07±0.31	47.11	71.93	3.942	8.82	0.00
Content-only channel gate	65.14±0.31	47.19	71.95	4.056	8.95	+0.07
Descriptor-conditioned channel gate	65.17±0.33	47.26	71.91	4.071	8.97	+0.10

**Table 11:** Three-seed PIR-SBFR ablation on DIOR. AP is mean ± sample SD; changes are relative to A0. A1 adds only degradation augmentation. A2 adds the scale-supervised visual route and P5 bypass. A3–A7 retain augmentation and the bypass but use only the named analytic or learned metadata route. A8 combines the visual residual with the analytic route, and A9 adds consistency training. GSD stays at its reference during paired training and clean evaluation. All accuracy values are percentages.

ID	Variant	AP	${\mathrm{AP}}_{S}$	${\mathrm{AP}}_{L}$	ΔAP
A0	Internal visual-only baseline	62.98±0.43	44.08	70.57	0.00
A1	+ degradation augmentation	63.37±0.45	44.65	70.84	+0.39
A2	+ scale supervision + P5 bypass	63.69±0.43	45.21	70.91	+0.71
A3	MTF/PSF-only analytic route	64.11±0.44	45.82	71.47	+1.13
A4	SNR-only analytic route	63.93±0.45	45.43	71.01	+0.95
A5	Combined analytic route (GSD at reference)	64.87±0.42	46.77	71.78	+1.89
A6	Metadata direct concatenation	63.98±0.42	45.62	71.18	+1.00
A7	Metadata-FiLM	64.40±0.42	46.21	71.46	+1.42
A8	Analytic prior + visual residual	65.17±0.42	47.18	71.98	+2.19
A9	+ degradation consistency (Full)	65.52±0.42	**47.61**	**72.19**	**+2.54**

Bold values identify the highest value in each metric column.

**Table 12:** Paired 2×2 ablation of scale supervision and the P5 structural bypass on DIOR. Cells are mean ± sample SD over seeds 2023–2025. The final row gives the paired factorial interaction from Equation (27). All values are percentages.

Configuration	Scale Supervision	P5 Bypass	AP	${\mathrm{AP}}_{S}$	${\mathrm{AP}}_{L}$
B0: shared baseline	No	No	63.37±0.45	44.65±0.45	70.84±0.32
BS: scale supervision only	Yes	No	63.57±0.44	45.10±0.46	70.73±0.32
BP: P5 bypass only	No	Yes	63.48±0.43	44.78±0.44	70.93±0.32
BSP: joint configuration	Yes	Yes	63.69±0.43	45.21±0.44	70.91±0.31
Paired interaction	–	–	+0.01±0.03	−0.02±0.02	+0.09±0.01

**Table 13:** Three-checkpoint mean within-set effects of metadata perturbation on the separate mixed-degradation DIOR control set. Correct pairing is the zero reference. Only percentage-point changes are reported to avoid conflation with clean-set AP.

Condition	ΔAP50	ΔAP	$\Delta {\mathrm{AP}}_{S}$	$\Delta {\mathrm{AP}}_{L}$
Correctly paired metadata	0.00	0.00	0.00	0.00
Multiplicative error ±10%	−0.29	−0.28	−0.37	−0.21
Multiplicative error ±20%	−0.91	−0.76	−1.20	−0.52
Multiplicative error ±50%	−1.75	−1.64	−2.43	−1.03
Missing 25%	−0.70	−0.61	−0.88	−0.37
Missing 50%	−1.21	−1.15	−1.65	−0.82
Missing 100%	−1.95	−1.80	−2.42	−1.31
Training-set mean metadata	−1.74	−1.67	−2.14	−1.11
Cross-image shuffled metadata	−2.36	−2.38	−3.24	−1.30

**Table 14:** Three-seed mean performance under degradation types held out from training and model selection. AP, ${\mathrm{AP}}_{S}$, and retention are percentages; ΔAP is expressed in percentage points.

Unseen Condition	Internal AP	PIR AP	ΔAP	PIR ${\mathrm{AP}}_{S}$	Retention
Defocus PSF	51.58	55.21	+3.63	38.92	84.26
Motion PSF	49.65	53.17	+3.52	36.68	81.15
Anisotropic PSF	50.14	54.09	+3.95	37.71	82.55
Speckle noise	52.38	55.52	+3.14	40.23	84.74
Stripe + read noise	51.84	54.80	+2.96	39.48	83.64
Unseen GSD 1.5×	56.71	59.53	+2.82	42.46	90.86
Unseen GSD 2.5×	46.70	51.86	+5.16	32.82	79.15
Unseen GSD 4×	33.95	40.74	+6.79	23.47	62.18
Joint degradation	29.60	36.97	+7.37	20.75	56.43
**Mean/worst**	**46.95/29.60**	**51.32/36.97**	**+4.37/+7.37**	**34.72**	**78.33**

Bold entries identify the summary row. Paired entries give the mean/lowest-AP condition; the gain after the slash is for that condition. The final two entries are means.

**Table 15:** Training-run uncertainty for PIR-SBFR Full versus the internal visual-only baseline. AP values are mean ± sample SD across ten runs. Difference statistics use seed-paired AP values and nine degrees of freedom. Changes and intervals are percentage points.

Dataset	Baseline AP	PIR-SBFR AP	Paired Change	95% *t*-Interval
DIOR	62.978±0.321	65.518±0.403	+2.540±0.318	[2.313, 2.767]
AI-TOD-v2	29.576±0.272	32.096±0.410	+2.520±0.378	[2.250, 2.790]

**Table 16:** Accuracy–efficiency comparison at 640×640 and batch size 1. Panels (**a**,**b**) report RTX 4090 resources and forward speed; this boundary excludes preprocessing, transfer, NMS, and serialization. The internal-baseline and PIR-SBFR AP values are ten-run means, the visual-demand/P5-preservation AP is a retained three-seed mean, and all resource and timing entries are single standardized profiles. Panels (**c**,**d**) report exported Jetson Orin NX measurements for the complete end-to-end boundary. The listed Jetson rate is 1000/p50, not independently measured sustained throughput.

**(a) Model resources**						
**Model**	**Params (M)**	**GFLOPs**	**Peak VRAM (GB)**		**FP16 Size (MB)**	
YOLOv11n	2.584	6.47	0.721		5.17	
Internal visual-only baseline	3.628	8.43	0.858		7.28	
Visual-demand + P5-preservation control	3.691	8.51	0.872		7.41	
**PIR-SBFR Full**	3.942	8.82	0.903		7.90	
**(b) Forward speed and accuracy**						
**Model**	**Latency (ms)**		**FPS**		**AP**	
YOLOv11n	2.347		426.1		57.31	
Internal visual-only baseline	3.079		324.8		62.98	
Visual-demand + P5-preservation control	3.117		320.8		63.69	
**PIR-SBFR Full**	3.313		301.8		**65.52**	
**(c) Jetson Orin NX end-to-end latency**						
**Model**	**Precision**	**AP**	**p50 (ms)**	**p95 (ms)**	**p99 (ms)**	**p50-Equiv. FPS**
Internal baseline	FP16	62.96	18.36	19.08	20.11	54.47
**PIR-SBFR**	FP16	**65.49**	19.55	20.47	21.63	51.15
Internal baseline	INT8	62.57	15.06	15.82	16.71	66.40
**PIR-SBFR**	INT8	**65.13**	16.03	16.91	17.84	62.38
**(d) Jetson operating measurements**						
**Model**	**Precision**	**Peak RAM (GB)**	**Power (W)**	**30 min Temperature**	**Engine Size (MB)**	
Internal	FP16	2.31	17.6	63.4 °C	8.3	
**PIR-SBFR**	FP16	2.47	18.2	65.1 °C	9.0	
Internal	INT8	1.92	16.2	61.8 °C	5.6	
**PIR-SBFR**	INT8	2.05	16.9	63.0 °C	6.1	

Bold model names identify PIR-SBFR. Bold AP values denote the highest AP in panel (**b**), or within each precision setting in panel (**c**).

## Data Availability

The image data analyzed in this study are publicly available. DIOR is available from the dataset authors at https://gcheng-nwpu.github.io/ (accessed on 11 September 2026), and AI-TOD-v2 is available at https://github.com/Chasel-Tsui/AI-TOD-v2 (accessed on 11 September 2026). All image-based experiments use only these public sources. Controlled observation-quality variants were generated from the public images using the transformations described in Section 3.5.3. Aggregate training-run and Jetson deployment measurements are reported in this article; the associated run summaries and hardware records are available from the corresponding author on reasonable request. The open-source PIR-SBFR implementation is available at https://github.com/Nopon-Knowledge/PIR-SBFR (accessed on 11 September 2026).

## Abbreviations

The following abbreviations are used in this manuscript:

AFPN	Asymptotic Feature Pyramid Network
AI-TOD-v2	Aerial Images Tiny Object Detection Dataset, version 2
AP	Average precision
AP50/75	AP at IoU thresholds of 0.50/0.75
AR	Average recall
ASFF	Adaptively Spatial Feature Fusion
BiFPN	Bidirectional Feature Pyramid Network
BN	Batch normalization
CI	Confidence interval
COCO	Common Objects in Context
CUDA	Compute Unified Device Architecture
DIOR	Dataset for Object Detection in Optical Remote Sensing Images
DRFB	Dilated Receptive Field Block
F1	Harmonic mean of precision and recall
FACH	Feature-Aware Coupled Head
FiLM	Feature-wise linear modulation
FP16	16-bit floating-point precision
FPN	Feature Pyramid Network
FPGA	Field-programmable gate array
FPS	Frames per second
GAP	Global average pooling
GFLOP	Giga floating-point operation
GPU	Graphics processing unit
GSD	Ground sampling distance
HSV	Hue–saturation–value
IoU	Intersection over union
INT8	8-bit integer precision
KL	Kullback–Leibler
MTF	Modulation transfer function
NMS	Non-maximum suppression
PAN	Path Aggregation Network
PIR-SBFR	Physical Imaging Reliability-Guided Scale-Biased Feature Reweighting
PSF	Point-spread function
RGB	Red–green–blue
SD	Sample standard deviation
SNR	Signal-to-noise ratio
VRAM	Video random-access memory
VT	Very tiny
YOLO	You Only Look Once
